# Relations between morphology, buoyancy and energetics of requiem sharks

**DOI:** 10.1098/rsos.160406

**Published:** 2016-10-26

**Authors:** Gil Iosilevskii, Yannis P. Papastamatiou

**Affiliations:** 1 Faculty of Aerospace Engineering, Technion, Haifa 32000, Israel; 2 Department of Biological Sciences, Florida International University, Miami, FL 33181, USA

**Keywords:** cost of transport, active metabolic rate, optimal swim speed, sharks

## Abstract

Sharks have a distinctive shape that remained practically unchanged through hundreds of millions of years of evolution. Nonetheless, there are variations of this shape that vary between and within species. We attempt to explain these variations by examining the partial derivatives of the cost of transport of a generic shark with respect to buoyancy, span and chord of its pectoral fins, length, girth and body temperature. Our analysis predicts an intricate relation between these parameters, suggesting that ectothermic species residing in cooler temperatures must either have longer pectoral fins and/or be more buoyant in order to maintain swimming performance. It also suggests that, in general, the buoyancy must increase with size, and therefore, there must be ontogenetic changes within a species, with individuals getting more buoyant as they grow. Pelagic species seem to have near optimally sized fins (which minimize the cost of transport), but the majority of reef sharks could have reduced the cost of transport by increasing the size of their fins. The fact that they do not implies negative selection, probably owing to decreased manoeuvrability in confined spaces (e.g. foraging on a reef).

## 1. Introduction

Within marine environments, sharks represent a wide range of upper and mid-level predators. They can be found in most marine habitats from coastal to pelagic and deep sea, and encompass a variety of feeding modes, including those specializing on marine mammals and filter-feeding [1]. These habitats also span a wide range of temperatures from arctic to tropical conditions. All sharks lack a swim bladder and therefore must generate lift either by retaining large amounts of low-density lipids (hydrostatic lift) or by generating flow of water over their fins (hydrodynamic lift). In spite of the lipids reserves, the majority of sharks are negatively buoyant and sink if they stop swimming (table 1).

**Table 1.** Nomenclature.

*b*	span of the pectoral fins
*c*_0_	root chord of a pectoral fin
*C*, *C*_+_, C_*_	cost of transport in general, cost of transport at $v=v_{+}$ and $v=v_{*}$
*C*_*f*_	friction coefficient
*C*_*D*_, *C*_*L*_	drag and lift coefficients based on reference area *S*
*C*_*D*0_	parasite (no lift) drag coefficient based on reference area *S*
*C*_*D*,max_	drag coefficient at the maximal lift coefficient
*C*_*L*,max_	maximal lift coefficient based on reference area *S*
*D*	hydrodynamic drag
*d*	maximal effective body diameter, $\sqrt{4S_{b}\text{/}\pi }$
*g*	acceleration of gravity
*K*	induced drag coefficient based on reference area *S*
*k*_*K*_	phenomenological constant in (3.7)
*k*_*m*_	prismatic coefficient of the shark's body defined in (3.1)
*k*_*P*_, *k*_*τ*_	phenomenological constants in (3.14)
*L*	hydrodynamic lift
*l*	pre-caudal (or fork) length
*l_t_*	total length
*m*	mass
*P*	active metabolic rate
*P*_0_	basic metabolic rate
*P*_+_, P_*_, *P*_min_	active metabolic rates at $v=v_{+}$, $v=v_{*}$ and $v=v_{\min}(0)$
*S*	general reference area, either *S*_*b*_ or *S*_*p*_
*S*_*b*_, *S*_*p*_	maximal cross-section area of the body; gross projected are of the pectoral fins
$S_{D0}$, $S_{D0}^{\left(b\right)}$	parasite drag area ($S_{D0}=SC_{D0}$), parasite drag area of the body
*s*	distal margin of a fin
*T*	thrust
*u*, $\bar{u}$	characteristic speed defined in (3.17); $\bar{u}=u\text{/}w$
v, $\bar{v}$	swim speed; $\bar{v}=v\text{/}w$
${\bar{v}}_{c}$, ${\bar{v}}_{2}$	dimensionless quantities defined in (4.14) and (4.21)
$v_{\min}(\gamma )$	minimal swim speed at angle *γ* relative to horizon
$v_{+}$,${\bar{v}}_{+}$	swim speed yielding the minimal active metabolic rate; ${\bar{v}}_{+}=v_{+}\text{/}w$
$v_{*}$, ${\bar{v}}_{*}$	swim speed yielding the minimal cost of transport; ${\bar{v}}_{*}=v_{*}\text{/}w$
*W*	weight of the shark in the water
*w*	characteristic speed defined in (3.18)
*x*	generic sensitivity parameter: *b*, *c*_0_, *d*, *l*, *β*, or *τ*
*α*	phenomenological parameter in (3.14); angle of attack in figure 1 (only)
*B*_*l*_	reduced excess buoyancy of the liver oils $B_{l}=(\rho -\rho_{l})\text{/}\rho$
*β*	reduced excess density $\beta =\text{( }\rho_{b}-\rho \text{) /}\rho$
*γ*	swim angle relative to horizon
*η*, *η*_*m*_	propulsion and muscle efficiencies
*ρ*, *ρ_l_*	density of water, effective density of the liver oils
*ρ_b_*	effective density of the shark
*τ*	body temperature
$\bar{\ldots }$	a reduced quantity

Being forced to swim continuously to generate hydrodynamic lift, sharks are faced with choices regarding their swim speed. As the swim speed increases, so does the metabolic cost, and the probability of a successful encounter with prey. In all cases, sharks—as other predators—probably select the swim speed that maximizes the difference between the energy obtained from prey and the energy spent searching for it. This speed depends on morphology and buoyancy, each affecting the hydrodynamic resistance, as well as on body temperature, which affects the basic metabolic rate [2,3]. Most species of sharks are ectothermic, so variations in body temperature reflect variations in the water temperature the shark resides in.

With a few exceptions, sharks evolved having similar (fusiform) basic body shape, but with considerable differences (some of which are ontogenetic) in the relative size of fins, relative body diameter and the amount and composition of lipids retained in the body [4–9]. In this study, we suggest a unified theory (theoretical framework) that can relate some of these differences with particular lifestyles and habitats, and can explain some of the ontogenetic differences as direct consequences of allometric scaling laws of swimming performance. It is based on general predictions of energetic costs of activity in sharks and swimming speeds that minimize these costs, and specific predictions of the influences of the most conspicuous morphological parameters, buoyancy and temperature on the energetic costs and on the respective optimal speeds.

The theory is presented in §§3 and 4; its few immediate conclusions ensue the developments of §3.6, 3.7, 4.7 and 5; overviewing discussion concludes the paper in §6. The data used in the analysis are presented in §2.

## 2. Underlying data

The ideal dataset for this study would have included tracking data (speed, depth, body temperature, water temperature and salinity), along with the respective morphological data (length, girth, fins dimensions), and in and out of water weights, for many individuals of different species. At present, no such dataset exists. The set compiled for this study (electronic supplementary material, S1, table S2) included 58 individuals from nine species of morphologically similar requiem sharks: *Carcharhinus obscurus*, *C. leucas*, *C. plumbeus*, *C. brevipinna*, *C. limbatus*, *C. falciformis*, *Negaprion brevirostris*, *Galeocerdo cuvier* and *Prionace glauca*, for which in and out of water weights were reported in [7,9]. Morphological data for these individuals were estimated based on relative dimensions reported in [4,5,10]. Hydrodynamic data were estimated from morphological data, using aircraft preliminary design tools [11] (electronic supplementary material, S1). We could evaluate the accuracy of these estimates, using wind tunnel measurements at relevant Reynolds numbers (electronic supplementary material, S2); they were accurate to within a few per cent.

## 3. Fundamentals

### 3.1. Lift and drag

Consider a negatively buoyant fish swimming at constant speed along a straight path, inclined at angle *γ* relative to horizon (positive when ascending). *ρ*, *v*, *g* and *m* are density of water, the swimming speed, the acceleration of gravity and the displaced mass of water, respectively. The latter can be expressed as

3.1 $$m=\rho S_{b}lk_{m},$$

where *l* and *S*_*b*_ are the (pre-caudal or fork) length of the fish and its maximal cross-section area and *k*_*m*_ is the prismatic coefficient—the ratio between the volume of a body and the volume of the minimal cylinder enclosing it; *k*_*m*_ ranges between 0.5 and 0.6 for most fish.

When swimming at constant speed, the hydrodynamic lift *L* and thrust *T* counterbalance drag^1^ *D* and weight *W*

3.2 T=D+Wsin⁡γ

and

3.3 L=Wcos⁡γ.

Hydrodynamic lift and drag are commonly expressed as

3.4 $$L=\frac{1}{2}\rho v^{2}SC_{L}$$

and

**Figure 1.**
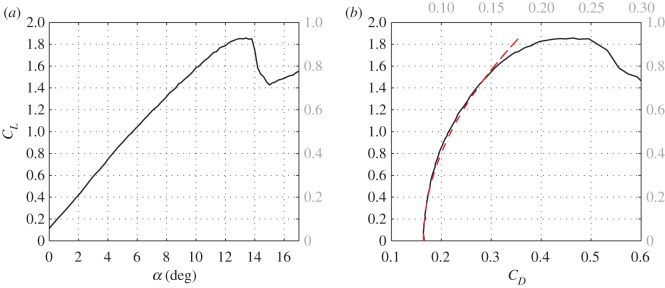
Lift and drag coefficients, *C*_*L*_ and *C*_*D*_, of a fictitious shark as measured in the wind tunnel at length-based Reynolds number of 2 × 10^6^. This particular shark has the same morphology as the great hammerhead (*Sphyrna mokarran*), except for the head which has been rounded to appear as a typical requiem shark. Details of the experiment can be found in electronic supplementary material, S2. In (*a*), *α* is the angle between the shark centreline and the swimming direction when pectoral fins are aligned with the centreline. Reference area is the maximal cross-section area of the body. In (*b*), the dotted line marks a curve-fitting parabola (3.6). In grey letters to the right of both figures and on the top of (*b*) are the corresponding values of lift and drag coefficients when the reference area is the gross projected area of the pectoral fins (which was twice the cross-section area of the body). Separation starts above *α *= 10°, where the curve-fitting parabola on (*b*) starts to deviate from the data, and develops into a full stall at *α *= 14°, where the lift coefficient drops.

3.5 $$D=\frac{1}{2}\rho v^{2}SC_{D},$$

where *S* is an arbitrary reference area, and *C*_*L*_ and *C*_*D*_ are the lift and drag coefficients. We assume that the lift is contributed mainly by the pectoral fins,^2^ and therefore, the lift coefficient depends mainly on the angle between the lifting surfaces (pectoral fins) and the flow (figure 1*a*). We also assume that the drag coefficient depends mainly on the lift coefficient with

3.6 $$C_{D}=C_{D0}+KC_{L}^{2},$$

where *C*_*D*0_ is the parasite (zero lift) drag coefficient, and

3.7 $$K=\frac{k_{K}}{\pi }\frac{S}{b^{2}}$$

is the induced drag coefficient. Here, *b* is the span of the pectoral fins, $k_{K}$ is a numerical factor accounting for increased flow separation from the surface of the fin with increasing angle of attack, for non-elliptical distribution of lift along the span and, to some extent, for the lift generated by other fins. *C*_*D*0_ depends on the geometry of the shark and (weakly) on the Reynolds number.^3^ When the reference area is chosen as the cross-section area of the body, typical value of *C*_*D*0_ for a 3 m shark swimming at 0.7 m s^−1^ is 0.17 (figure 1*b*); typical value of $k_{K}$ is 1.5. The product

3.8 $$S_{D}=SC_{D},$$

is referred to as the ‘drag area’. It has the advantage of being independent of the choice of the reference area. A particular case of (3.8) is $S_{D0}=SC_{D0}$.

Submerged weight of the shark, *W*, can be expressed in terms of the excess density parameter, *β*

3.9 W=mgβ.

**Figure 2.**
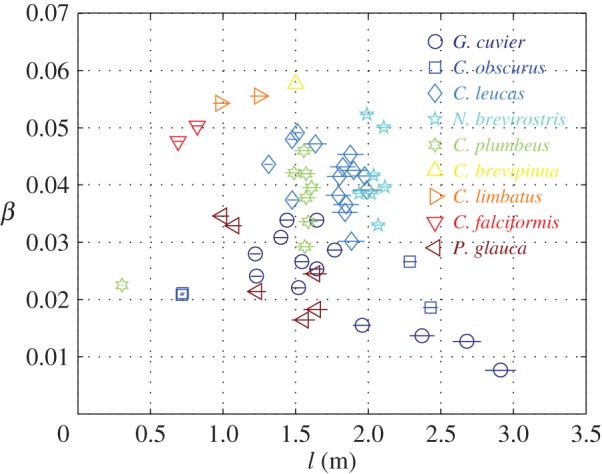
Excess density parameter *β* of 58 individuals from nine species of requiem sharks. Horizontal bars mark the uncertainty range. Data are based on [7,9,10]. Numerical values underlying this figure can be found in electronic supplementary material, S1, table S2*a*.

For most sharks, *β* varies between 0% and 6% (figure 2).

In combination with (3.4), the balance of forces in the direction normal to the direction of swimming (3.3) can be used to define either the lift coefficient

3.10 $$C_{L}=\frac{2W\cos\gamma }{\rho Sv^{2}},$$

needed to counteract weight at a given swimming speed, or the swimming speed

3.11 $$v^{2}=\frac{2W\cos\gamma }{\rho SC_{L}},$$

needed to counteract weight at a given lift coefficient. Similarly, in combination with (3.5) and (3.10), the balance of forces in the direction of swimming (3.2) can be used to define either the thrust needed to sustain speed or the sustained speed for a given thrust. Note that when descending idle (*T* = 0),

3.12 $$\tan\gamma =-\frac{C_{D}}{C_{L}}\;\text{and}\;v^{2}=\frac{2W}{\rho S\sqrt{C_{L}^{2}+C_{D}^{2}}},$$

by (3.2) and (3.5).

### 3.2. Active metabolic rate

Active metabolic rate is defined here as the total amount of ATP used by the fish per unit time

3.13 $$P=P_{0}+\frac{Tv}{\eta \eta_{m}}.$$

It comprises the standard metabolic rate, *P*_0_, and the cost of activity, $Tv\text{/}\eta_{m}\eta$. *η*_*m*_ is the chemomechanical efficiency of the muscles (the mechanical work done per mole ATP) and *η* is the hydrodynamic propulsion efficiency. Both efficiencies are assumed independent of the shark's morphology and swimming conditions; their typical values are 24 J per mmol ATP [18] and 0.7 [19], respectively.^4^^,^^5^^,^^6^ *P*_0_ is approximated with

3.14 $$P_{0}=k_{P}m^{\alpha }{\text{e}}^{-k_{\tau }\text{/}\tau },$$

where *τ* is the absolute body temperature, and *k_P_*, α and *k_τ_* are certain phenomenological parameters. Their typical values are 127 mol ATP per s·kg^α^, 0.8, and 5020°K, respectively [23], but there can be interspecific differences in these parameters [24].

In a glide, *T* = 0, and the active metabolic rate equals the standard metabolic rate, *P*_0_. In what follows, however, we assume that the shark swims at constant depth and speed; consequently *T* = *D* by (3.2), and

3.15 $$\begin{array}{rl} P & =P_{0}+\frac{\rho S}{2\eta \eta_{m}}{\left(\frac{2W}{\rho S}\right)}^{3\text{/}2}\left(\frac{C_{D0}}{C_{L}^{3/2}}+KC_{L}^{1\text{/}2}\right)\\ & =P_{0}+\frac{\rho S}{2\eta \eta_{m}}\left(C_{D0}v^{3}+\frac{K}{v}{\left(\frac{2W}{\rho S}\right)}^{2}\right) \end{array}$$

by (3.6), (3.5), (3.10) and (3.11). Equation~(3.15) can be rewritten as

3.16 $$P=P_{0}\left(1+\frac{1}{2}\frac{v^{3}}{w^{3}}+\frac{1}{2}\frac{u^{4}}{w^{3}v}\right),$$

where

3.17 $$u={\left(\frac{K}{C_{D0}}\right)}^{1\text{/}4}{\left(\frac{2W}{\rho S}\right)}^{1/2}={\left(\frac{k_{K}}{\pi b^{2}S_{D0}}\right)}^{1/4}{\left(\frac{2mg\beta }{\rho }\right)}^{1/2}$$

and

**Figure 3.**
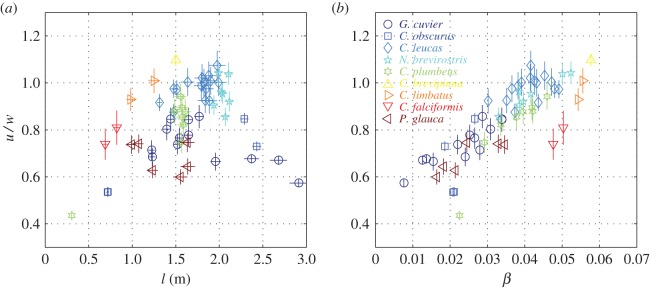
Estimated values of the velocities ratio *u*/*w* for the individual sharks from electronic supplementary material, S1, table S2*b*. The ratio is presented against the pre-caudal length (*a*) and against the relative excess density (*b*). The lowest point belongs to a pup of *C. plumbeus.* Crosses mark the uncertainty range. Note that as *β* increases, the buoyancy decreases.

3.18 $$w={\left(\frac{\eta \eta_{m}P_{0}}{\rho S_{D0}}\right)}^{1\text{/}3}$$

are a pair of characteristic velocity scales; their physical meaning becomes clear in §3.3. The ratio *u*/*w* is a variable parameter but, in general, can be considered an order 1 quantity (figure 3).

If there were no constraints, then the minimal active metabolic rate,

3.19 $$P_{+}=P_{0}\left(1+\frac{2}{3^{3/4}}\frac{u^{3}}{w^{3}}\right),$$

would have been obtained at

3.20 $$v_{+}=\frac{u}{3^{1/4}},$$

the solution of the equation

3.21 $$\frac{\partial P}{\partial v}=0.$$

For a neutrally buoyant fish (*β* = 0), *v*_+_ = *u* = 0 and *P*_+_ = *P*_0_. For a non-neutrally buoyant fish (β≠0), the minimal speed is typically limited by stall of the pectoral fins, and minimal active metabolic rate is obtained at the lowest swimming speed, *v*_min_ rather than at *v*_+_ (see section 3.6).

### 3.3. Cost of transport

The cost of transport *C* is defined as the energy used per distance travelled

3.22 $$C=\frac{P}{v}.$$

Substituting (3.16) it takes on the form

3.23 $$C=\frac{P_{0}}{w}\left(\frac{w}{v}+\frac{1}{2}\frac{u^{2}}{w^{2}}\left(\frac{v^{2}}{u^{2}}+\frac{u^{2}}{v^{2}}\right)\right)=\frac{P_{0}}{w}\left(\frac{w}{v}+\frac{1}{2}\frac{v^{2}}{w^{2}}+\frac{1}{2}\frac{u^{4}}{w^{2}v^{2}}\right).$$

Minimal cost is obtained at the swimming speed, say v_*_, at which

3.24 $$\frac{\partial C}{\partial v}=0.$$

This condition leads to the equation

3.25 $$w^{3}v_{*}-v_{*}^{4}+u^{4}=0.$$

Its solution is

**Figure 4.**
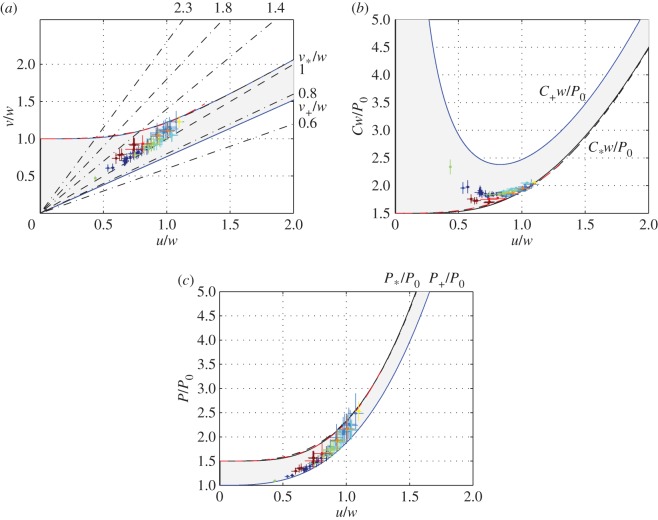
Minimal cost of transport (*b*), minimal active metabolic rate (*c*) and the swimming speed at which they are achieved (*a*) as functions of *u/w*. Exact solution is marked blue; approximations of the fourth and fifth rows of table 2 are marked dashed black and dashed red. Grey area marks the range between the minimal active metabolic rate and the minimal cost of transport. The slope of the straight dash-dotted lines in (*a*) is indicated to the right of each line. The range above steepest line is where having large fins is detrimental; below it is where having large fins is incremental. Crosses mark the estimated minimal swim speed for the individual sharks from electronic supplementary material, S1, table S2*b*.

3.26 $$v_{*}=w\left(1+\frac{1}{3}\frac{u^{4}}{w^{4}}+\cdots \right)$$

when *u *→ 0, and $v_{*}=u(1+(1/4){\left(w/u\right)}^{3}+\cdots )$ when *w* → 0. It lacks a closed-form analytical solution for $v_{*}$ in between these two extremes, but interpolating formulae in the last two rows of table 2 offer very good fits (figure 4). For future reference, we note that $v_{*}>\max(u,w)$. This conjecture is apparent in figure 4*a*; it can be obtained formally by rearranging (3.25) as $v_{*}=w+u^{4}{\left(v_{*}(v_{*}^{2}+v_{*}w+w^{2})\right)}^{-1}$ or as $v_{*}=u+w^{3}v_{*}{\left(v_{*}^{3}+v_{*}^{2}w+v_{*}w^{2}+w^{3}\right)}^{-1}$. Thus, *w* and *u* are the speeds that minimize the cost of transport in the limits when the buoyancy (and hence the energetic cost of generating hydrodynamic lift) is very small and when the basic metabolic rate (the energetic cost of living) is very small, respectively. Typical values of $v_{*}$ can be found in electronic supplementary material, S1, table S2*b*.

**Table 2.** Sustained performance parameters. In all expressions, an overbar denotes a reduced speed: $\bar{u}=u\text{/}w$,${\bar{v}}_{\min}(\gamma )=v_{\min}(\gamma )\text{/}w$, ${\bar{v}}_{+}=v_{+}\text{/}w$ and ${\bar{v}}_{*}=v_{*}\text{/}w$. Reference equations for the first row are (3.34), (3.20), (3.25), (3.19), (3.28) and (3.27), respectively.

type	${\bar{v}}_{\min}(0)$	${\bar{v}}_{+}$	$P_{+}\text{/}P_{0}$	${\bar{v}}_{*}$	$P_{*}\text{/}P_{0}$	$C_{*}w\text{/}P_{0}$
exact	$\bar{u}{\left(\frac{C_{D0}}{KC_{L,\max}^{2}}\right)}^{1/4}$	$\frac{\bar{u}}{3^{1/4}}$	$1+\frac{2{\bar{u}}^{3}}{3^{3/4}}$	solution of ${\bar{v}}_{*}-{\bar{v}}_{*}^{4}+{\bar{u}}^{4}=0$	$1+\frac{{\bar{v}}_{*}^{3}}{2}+\frac{{\bar{u}}^{4}}{2{\bar{v}}_{*}}$	$\frac{1}{{\bar{v}}_{*}}+\frac{{\bar{v}}_{*}^{2}}{2}+\frac{{\bar{u}}^{4}}{2{\bar{v}}_{*}^{2}}$
series, $\bar{u}\ll 1$				1+1∫3∫u¯4+⋯	$\frac{3}{2}+{\bar{u}}^{4}+\cdots$	$\frac{3}{2}+\frac{1}{2}{\bar{u}}^{4}+\cdots$
series, $\bar{u}\gg 1$				u¯+1∫4∫u¯2+⋯	${\bar{u}}^{3}+\frac{5}{4}+\cdots$	${\bar{u}}^{2}+\frac{1}{\bar{u}}+\cdots$
best fit, $\bar{u}\in (0,\infty )$				6+3u¯11/3+8u¯7∫6+8u¯6∫	$\frac{6+10{\bar{u}}^{11/3}+5{\bar{u}}^{7}}{4+5{\bar{u}}^{4}}$	$\frac{9+5{\bar{u}}^{11/3}+3{\bar{u}}^{7}}{6+3{\bar{u}}^{5}}$
best fit, $\bar{u}\in (0,1.3)$				1+3∫14∫u¯3	$\frac{3}{2}+\frac{4}{5}{\bar{u}}^{7/2}$	$\frac{3}{2}+\frac{2}{5}{\bar{u}}^{3}$

The minimal cost of transport and the respective metabolic rate are

3.27 $$C_{*}=\frac{P_{0}}{w}\left(\frac{w}{v_{*}}+\frac{1}{2}\frac{v_{*}^{2}}{w^{2}}+\frac{1}{2}\frac{u^{4}}{w^{2}v_{*}^{2}}\right)$$

and

3.28 $$P_{*}=C_{*}v_{*}$$

by (3.22) and (3.23). Because $v_{*}$ lacks a closed-form expression relating it with *u* and *w*, so does C_*_ and P_*_. They can be approximated with

3.29 $$C_{*}=\frac{3}{2}\frac{P_{0}}{w}\left(1+\frac{1}{3}\frac{u^{4}}{w^{4}}+\cdots \right)$$

and

3.30 $$P_{*}=\frac{3}{2}P_{0}\left(1+\frac{2}{3}\frac{u^{4}}{w^{4}}+\cdots \right)$$

when say, *u*/*w* < 0.6; more elaborate approximations can be found in table 2.

The terms in the parentheses on the right-hand side of (3.26), (3.29) and (3.30) manifest the difference between negatively and neutrally buoyant fishes (for which *u* = 0). Negatively buoyant fish have to swim faster than similarly shaped neutrally buoyant ones, and their cost of transport and active metabolic rate is higher. In fact, estimated optimal swimming speeds of *C. leucas*, *C. limbatus*, *C*. *brevipinna* and *N. breviostris* are up to 30% higher than what they would have been if these sharks were neutrally buoyant; respective costs of transport are up to 40% higher (see electronic supplementary material, S1, table S2*b*).

### 3.4. The speed ratio

Choosing *w* as a unit of speed, and the basic metabolic rate *P*_0_ as a unit of power, all reduced performance characteristics—the minimal active metabolic rate *P*_+_/*P*_0_, the minimal cost of transport $C_{*}w\text{/}P_{0}$ and the swimming speeds, $v_{+}\text{/}w$ and $v_{*}\text{/}w$, at which they are obtained—become dependent on a single parameter, $\bar{u}=u\text{/}w$. All four increase with $\bar{u}$ and hence, in many cases, energy expenditure of a shark can be reduced by making $\bar{u}$ small.

Expression for $\bar{u}$,

3.31 $$\begin{array}{rl} \frac{u}{w} & ={\left(\frac{4k_{K}}{\pi }\right)}^{1/4}{\left(\frac{mg\beta }{b}\right)}^{1/2}\frac{1}{{\left(\eta \eta_{m}P_{0}\right)}^{1/3}}{\left(\frac{S_{D0}}{\rho^{2}}\right)}^{1/12}\\ & ={\left(\frac{4k_{K}}{\pi }\right)}^{1/4}{\left(\frac{g\beta }{b}\right)}^{1/2}\frac{m^{1/2-\alpha /3}e^{k_{\tau }/3\tau }}{{\left(\eta \eta_{m}k_{P}\right)}^{1/3}}{\left(\frac{S_{D0}}{\rho^{2}}\right)}^{1/12}, \end{array}$$

follows by (3.17), (3.18) and (3.14). Because $m\propto l^{3}$, $S_{D0}\propto l^{2}$ (see footnote 2) and b∝l, we may expect $\bar{u}\propto {\left(\beta l\text{/}b\right)}^{1/2}l^{7/6-\alpha }e^{k_{\tau }/3\tau }$ or, what is equivalent, $\bar{u}\propto {\left(\beta l\text{/}b\right)}^{1/2}m^{7/18-\alpha /3}e^{k_{\tau }/3\tau }$; the powers with *l* and *m* are positive (0.36 and 0.12, respectively). Typical values of *u*/*w* can be found in figure 3; they do not exceed 1.1 for all individuals on our list, and do not exceed 0.8 for the two pelagic (*P. glauca* and *C. falciformis*) and the two ‘cosmopolitan’ (*C. obscurus* and *G. cuvier*) species included thereat. $\bar{u}$ can be reduced mainly by decreasing the negative buoyancy *β*, by increasing the span of the pectoral fins *b*/*l*, and by decreasing the mass. It can also be reduced by increasing the body temperature, but the resulting increase in the standard metabolic rate more than offsets the beneficial effect of reducing the value of $\bar{u}$ .

### 3.5. Energy balance

If prey is uniformly distributed along the swimming path, and the energy intake of the shark is directly proportional to the amount of prey encountered en route, the energy balance of a shark—the difference between energy gained *E*_in_ and the energy spent *E*_out_—can be expressed (with help of (3.22)) as

3.32 $$\Delta E=E_{\mathrm{in}}-E_{\mathrm{out}}=\int_{0}^{T}(ev-P)\;\text{d}t=\int_{0}^{T}(e-C)v\;\text{d}t=\int_{0}^{X}\left(e-C\right)\;\text{d}x,$$

where *X* is the distance swum at speed *v*, *T* is the swimming duration and *e* is a certain coefficient reflecting the prey density and the probability of its capture. Minimizing the cost of transport, *C*, maximizes the energy gain, irrespective of *e* [25].

If, however, the amount of food encountered by the shark is independent of the volume of water searched during swimming, but depends only on time, then the energy balance becomes

3.33 $$\Delta E=E_{\mathrm{in}}-E_{\mathrm{out}}=\int_{0}^{T}(e^{\prime }-P)\;\text{d}t,$$

where *e*′ reflects the prey encounter rate and the probability of its capture. Minimizing the active metabolic rate, *P*, maximizes the energy gain, irrespective of *e*′.

Realistic scenarios are bounded between these two extremes, suggesting that a shark probably swims between *v*_+_, the speed at which its active metabolic rate is minimal, and v_*_, the speed at which its cost of transport is minimal (this conjecture is assessed in §3.7); its active metabolic rate varies between *P*_+_ and P_*_ (figure 4). The prerequisite to this analysis is that v_*_ and *v*_+_ exceed a certain minimal swimming speed.

### 3.6. Minimal swimming speed

From a hydrodynamic perspective, the minimal swimming speed is the lowest speed at which the forces acting on the shark can be balanced. It is an immediate consequence of the existence of the upper bound $C_{L,\max}$ on the lift coefficient (figure 1*a*); in fact

3.34 $$v_{\min}(\gamma )={\left(\frac{2W\cos\gamma }{\rho SC_{L,\max}}\right)}^{1/2}=u{\left(\frac{C_{D0}}{KC_{L,\max}^{2}}\right)}^{1/4}{\left(\cos\gamma \right)}^{1/2},$$

at powered ascent or descent, and

3.35 $$v_{\min}(-\gamma_{0})=u{\left(\frac{C_{D0}}{KC_{L,\max}^{2}}\right)}^{1/4}{\left(\frac{C_{L,\max}^{2}}{C_{L,\max}^{2}+C_{D,\max}^{2}}\right)}^{1/4},$$

when gliding at angle $\gamma_{0}=\tan^{-1}(C_{D,\max}\text{/}C_{L,\max})$ with zero thrust; *C*_*D*,max_ is the drag coefficient at $C_{L}=C_{L,\max}$. Equation (3.34) follows from (3.11) and (3.17); equation (3.35) follows from (3.12). Because $C_{D,\max}^{2}$ is invariably small relative to $C_{L,\max}^{2}$ (figure 1), the minimal glide speed $v_{\min}(-\gamma_{0})$ and the minimal speed at constant depth $v_{\min}(0)$ are hardly different. At the same time, the minimal speed in vertical ascent $v_{\min}(\pi \text{/}2)$ is identically zero. Typical values of ${\left(C_{D0}\text{/}KC_{L,\max}^{2}\right)}^{1/4}$ range between 1 and 1.4 for all practical combinations of morphological parameters.^7^

To exploit the minimal active metabolic rate when swimming at constant depth, *v*_+_ should exceed $v_{\min}(0)$. It implies

3.36 $${\left(\frac{3C_{D0}}{KC_{L,\max}^{2}}\right)}^{1/4}<1,$$

by (3.34) and (3.20). This condition cannot be satisfied with any admissible set of morphological parameters (see the preceding paragraph), and no shark on our list can exploit the minimal active metabolic rate when swimming at constant depth (figure 4). Consequently, the lowest active metabolic rate when swimming at constant depth, $\underset{C_{L}\leq C_{L,\max}}{\min}P$, is achieved at the minimal swimming speed, $v_{\min}(0)$. Given that the difference between $v_{\min}(0)$ and *v*_+_ is small, the difference between $\underset{C_{L}\leq C_{L,\max}}{\min}P$ and $P_{+}$,

3.37 $$\frac{\underset{C_{L}\leq C_{L,\max}}{\min}P-P_{+}}{P_{0}}=3^{3/4}\frac{u}{w}{\left(\frac{v_{\min}(0)-v_{+}}{w}\right)}^{2}+\ldots$$

is also small (figure 4*c*). Nonetheless, because $\underset{C_{L}\leq C_{L,\max}}{\min}P>P_{+}$, minimizing the active metabolic rate was not *the* evolutionary objective with any of these species.

To exploit the minimal cost of transport when swimming at constant depth, v_*_ should exceed $v_{\min}(0)$. It implies

3.38 $$v_{\min}^{4}(0)-w^{3}v_{\min}(0)<u^{4},$$

by (3.25), which after some rearrangement, can be recast as

3.39 $$\frac{v_{\min}^{4}(0)}{u^{4}}-1<\frac{w^{3}}{u^{3}}\frac{v_{\min}(0)}{u}.$$

At the same time

3.40 $$\frac{v_{\min}^{4}(0)}{u^{4}}=\frac{C_{D0}}{KC_{L,\max}^{2}},$$

by (3.34); whence (3.39) sets un upper bound on the speed ratio

3.41 $$\frac{u}{w}<{\left(\frac{C_{D0}}{KC_{L,\max}^{2}}\right)}^{1/12}{\left(\frac{C_{D0}}{KC_{L,\max}^{2}}-1\right)}^{-1/3}.$$

For the same combinations of morphological parameters as those listed in footnote 7, the right-hand side of (3.41) ranges between 0.8 and 2. The left-hand side varies with buoyancy and body temperature, as well as with basic morphological parameters (see above), and is, in general, an order 1 quantity. Consequently, (3.41) is not automatically satisfied, and buoyancy and body temperature have to be coordinated with morphological parameters to allow a shark to exploit its minimal cost of transport. In particular, because $u\text{/}w\propto {\left(\beta l\text{/}b\right)}^{1/2}l^{7/6-\alpha }e^{k_{\tau }/3\tau }$ (see the paragraph following (3.31)), inequality (3.41) implies that large sharks (large *l*) and/or ectothermic sharks residing in cold water (small *τ*) must also have small negative buoyancy (small *β*) and/or large pectoral fins. Examples include the basking shark *Cetorhinus maximus* [9], the Portuguese dogfish *Centroscymnus coelolepis* [26], and the six-gill shark *Hexanchus griseus* [27].^8^ Some sharks exhibit ontogenetic increase in hepatosomatic index (proportional mass of the liver), which is inversely correlated with the value of *β*. Examples include the oceanic whitetip shark *C*. *longimanus* [6], the dusky shark *C*. *obscurus* [28] and the tiger shark *G. cuvier* [7]. Some sharks exhibit an ontogenetic increase in the span of the pectoral fins; examples include the bull shark *C. leucas* and dusky shark [5].

### 3.7. Optimal swimming speed

It was predicted in §§3.5 and 3.6 that under most circumstances, the optimal swimming speed of the shark is bounded between the larger of *v*_+_ and $v_{\min}(0)$, and v_*_. Reliable corroboration of this conjecture is complicated by the fact that average speed measurements are commonly cited without the necessary complementary data, which includes length, mass (or girth), temperature, span of the pectoral fins and buoyancy. Moreover, many of these measurements were made immediately after having released the shark, and hence may not reflect its natural behaviour [29]. Notwithstanding these caveats, reference [30] cites voluntary swimming speeds of two bull sharks and one sandbar shark *C. plumbeus* in a large water tank. The bull sharks were 2 and 2.3 m long, the sandbar shark was 2.1 m long (total length). They swam in 26°C water with average speeds of 0.72, 0.62 and 0.64 m s^−1^, respectively, accelerating and decelerating a few hundredth m s^−1^ about these values. Referring to electronic supplementary material, S1, tables S2*a* and S2*b*, v_*_ for comparably sized bull sharks (sharks 5,8,13 and 15) is between 0.67 and 0.78 m s^−1^, depending on buoyancy and morphology; v_*_ for comparably sized sandbar sharks (sharks 1–4,6 and 7) is between 0.62 and 0.74 m s^−1^. For both species, *v*_+_ is roughly 0.28 m s^−1^ smaller than v_*_.

Reference [31] cites average swimming speeds of three blue sharks *P. glauca*, tracked over the period of a few days (sharks 16, 22 and 23). With body temperatures of about 18°C, the three sharks, measuring 2.2, 2.7 and 2.6 m (fork length) averaged 0.48, 0.4 and 0.44 m s^−1^. There are no comparably sized sharks on our list, but the optimal speeds can be estimated based on the same formulae that underlay table S2 in electronic supplementary material, S1. With *β* = 0.02, and depending on the length of the pectoral fins and body mass, they yield v_*_ between 0.55 and 0.6 m s^−1^ for the two larger sharks, and between 0.52 and 0.56 for the smaller one; *v*_+_ is 0.27 m s^−1^ smaller than v_*_.

For the two bull sharks and the three blue sharks, we predict *v*_min_ (0) between 0.13 and a few hundredth m s^−1^ smaller than the respective v_*_, whereas for the sandbar shark, we predict it is between 0.2 and 0.08 m s^−1^ smaller. In other words, there are possible combinations of morphological parameters and buoyancy for which *v*_min_ (0) exceeds the observed swimming speed. *v*_min_ (0) is extremely sensitive to buoyancy (it vanishes with *β*), and hence obtaining unrealistic *v*_min_ (0) demonstrates the importance of having the ideal dataset mentioned in §2, as well as the importance of coordination between morphological parameters and buoyancy.

## 4. Derivatives

### 4.1. Preliminaries

Sustained performance of a shark is characterized mainly by the active metabolic rate, the cost of transport and the speed at which the minimal cost of transport is achieved. Essentially, there are six major morphological parameters affecting the sustained performance: length, *l*; span and chord of the pectoral fins, *b* and *c*_0_; body diameter, *d*; buoyancy, *β* and body temperature, *τ*. The first three can be considered an evolutionary adaptation; the next two also depend on an individual's body condition; the last two also depend on the habitat the animal uses. Sensitivity of the sustained performance to variations in these parameters is manifested in the partial derivatives computed below.

Quite generally, if *x* denotes one of the independent parameters, namely *β*, *b*, *c*_0_, *d*, *l* and *τ*, we can write a series of logarithmic derivatives

4.1 $$\frac{x}{v_{*}}\frac{\partial v_{*}}{\partial x}=\frac{1}{4v_{*}^{4}-w^{3}v_{*}}\left(4u^{4}\frac{x}{u}\frac{\partial u}{\partial x}+3w^{3}v_{*}\frac{x}{w}\frac{\partial w}{\partial x}\right)$$

and

4.2 $$\frac{x}{C_{*}}\frac{\partial C_{*}}{\partial x}=\frac{v_{*}}{C_{*}}\frac{\partial C_{*}}{\partial v_{*}}\frac{x}{v_{*}}\frac{\partial v_{*}}{\partial x}+\frac{P_{0}}{C_{*}}\frac{\partial C_{*}}{\partial P_{0}}\frac{x}{P_{0}}\frac{\partial P_{0}}{\partial x}+\frac{u}{C_{*}}\frac{\partial C_{*}}{\partial u}\frac{x}{u}\frac{\partial u}{\partial x}+\frac{w}{C_{*}}\frac{\partial C_{*}}{\partial w}\frac{x}{w}\frac{\partial w}{\partial x},$$

and, given *v*,

4.3 $$\frac{x}{P}\frac{\partial P}{\partial x}=\frac{x}{C}\frac{\partial C}{\partial x}=\frac{P_{0}}{P}\frac{\partial P}{\partial P_{0}}\frac{x}{P_{0}}\frac{\partial P_{0}}{\partial x}+\frac{u}{P}\frac{\partial P}{\partial u}\frac{x}{u}\frac{\partial u}{\partial x}+\frac{w}{P}\frac{\partial P}{\partial w}\frac{x}{w}\frac{\partial w}{\partial x}.$$

They follow from (3.25), (3.22) and (3.16). Being inherently dimensionless, logarithmic derivatives offer both simplicity of the final expressions and a convenient interpretation of the result. For example, the relative change in the cost of transport, ΔC_*_/C_*_, owing to a (small) relative change Δ*x*/*x* in an independent parameter, is

4.4 $$\frac{\Delta C_{*}}{C_{*}}=\left(\frac{x}{C_{*}}\frac{\partial C_{*}}{\partial x}\right)\frac{\Delta x}{x},$$

The logarithmic derivative $\left(x\text{/}C_{*})(\partial C_{*}\text{/}\partial x\right)$ serves as an amplification factor between Δ*x*/*x* and $\Delta C_{*}\text{/}C_{*}$; with $\left(x\text{/}C_{*})(\partial C_{*}\text{/}\partial x\right)$ = 0.1, say, a 10% change in *x* yields 1% change in C_*_.

The first term in (4.2) vanishes by (3.24). Substituting (3.23) for C_*_ in (4.3), and (3.16) for *P* in (4.3) yields

4.5 $$\frac{x}{C_{*}}\frac{\partial C_{*}}{\partial x}=\frac{x}{P_{0}}\frac{\partial P_{0}}{\partial x}+\frac{1}{2w^{3}v_{*}+v_{*}^{4}+u^{4}}\left(4u^{4}\frac{x}{u}\frac{\partial u}{\partial x}-3(v_{*}^{4}+u^{4})\frac{x}{w}\frac{\partial w}{\partial x}\right)$$

and

4.6 $$\frac{x}{P}\frac{\partial P}{\partial x}=\frac{x}{C}\frac{\partial C}{\partial x}=\frac{x}{P_{0}}\frac{\partial P_{0}}{\partial x}+\frac{1}{2w^{3}v+v^{4}+u^{4}}\left(4u^{4}\frac{x}{u}\frac{\partial u}{\partial x}-3(v^{4}+u^{4})\frac{x}{w}\frac{\partial w}{\partial x}\right).$$

Note that because $\partial C_{*}\text{/}\partial v_{*}=0$ by (3.24),

4.7 $$\frac{x}{C_{*}}\frac{\partial C_{*}}{\partial x}={\left(\frac{x}{C}\frac{\partial C}{\partial x}\right)}_{v=v_{*}};\;$$

this equivalence is manifested in (4.5) and (4.6).

Essentially, there are three primitive derivatives that are required to find all the others: $\frac{x}{P_{0}}\frac{\partial P_{0}}{\partial x}$, $\frac{x}{u}\frac{\partial u}{\partial x}$ and $\frac{x}{w}\frac{\partial w}{\partial x}$. The last two,

4.8 $$\frac{x}{u}\frac{\partial u}{\partial x}=\frac{1}{2}\frac{x}{m}\frac{\partial m}{\partial x}+\frac{1}{2}\frac{x}{\beta }\frac{\partial \beta }{\partial x}-\frac{1}{2}\frac{x}{b}\frac{\partial b}{\partial x}-\frac{1}{4}\frac{x}{S_{D0}}\frac{\partial S_{D0}}{\partial x}$$

and

4.9 $$\frac{x}{w}\frac{\partial w}{\partial x}=\frac{1}{3}\frac{x}{P_{0}}\frac{\partial P_{0}}{\partial x}-\frac{1}{3}\frac{x}{S_{D0}}\frac{\partial S_{D0}}{\partial x},$$

follow from (3.17) and (3.18); we assume that the remaining parameters, ρ, $k_{K}$, η and $\eta_{m}$ are, essentially, constants. In turn, *P*_0_ is commonly considered a function of mass and body temperature *τ* only, and hence

4.10 $$\frac{x}{P_{0}}\frac{\partial P_{0}}{\partial x}=2\alpha \frac{x}{m}\frac{\partial m}{\partial x}+\frac{k_{\tau }}{\tau }\frac{x}{\tau }\frac{\partial \tau }{\partial x}.$$

Explicit expressions for all pertinent derivatives are summarized in table 3; the underlying derivations and a few comments follow in §§4.2–4.8.

**Table 3.** Sensitivity derivatives. In all expressions, an overbar denotes a reduced quantity; in particular, $\bar{u}=u\text{/}w$, $\bar{v}=v\text{/}w$ and ${\bar{v}}_{*}=v_{*}\text{/}w$. ${\bar{v}}_{c}$ and ${\bar{v}}_{2}$ have been defined in (4.14) and (4.21); ${\bar{S}}_{D0}^{\left(b\right)}$ has been defined in (4.28). Expressions in the second and the fourth columns have been modified with the help of (3.25). If increasing the diameter does not increase the basic metabolic rate, *α* in the fifth row should be set to zero. Expressions in the third and fifth columns are approximations of the respective expressions to their lefts under the assumption that ${\bar{v}}_{c}^{4}\gg 1$ and $u^{3}\ll w^{3}$.

	$\frac{x}{v_{*}}\frac{\partial v_{*}}{\partial x}$		$\frac{x}{C_{*}}\frac{\partial C_{*}}{\partial x}$		$\frac{x}{C}\frac{\partial C}{\partial x}$ or $\frac{x}{P}\frac{\partial P}{\partial x}$
*x*	exact	approximation	exact	approximation	exact
*β*	$\frac{2{\bar{u}}^{4}}{3{\bar{v}}_{*}+4{\bar{u}}^{4}}$	2∫3∫u¯4	$\frac{2{\bar{u}}^{4}}{3{\bar{v}}_{*}+2{\bar{u}}^{4}}$	$\frac{2}{3}{\bar{u}}^{4}$	$\frac{2{\bar{u}}^{4}}{2\bar{v}+{\bar{v}}^{4}+{\bar{u}}^{4}}$
*τ*	$\frac{k_{\tau }}{\tau }\frac{{\bar{v}}_{*}}{3{\bar{v}}_{*}+4{\bar{u}}^{4}}$	kτ∫3τ∫	$\frac{k_{\tau }}{\tau }\frac{2{\bar{v}}_{*}}{3{\bar{v}}_{*}+2{\bar{u}}^{4}}$	2kτ∫3τ∫	$\frac{k_{\tau }}{\tau }\frac{2\bar{v}}{2\bar{v}+{\bar{v}}^{4}+{\bar{u}}^{4}}$
*b*	$-\frac{2}{{\bar{v}}_{c}^{4}}\frac{{\bar{v}}_{*}+{\bar{u}}^{4}({\bar{v}}_{c}^{4}+1)}{3{\bar{v}}_{*}+4{\bar{u}}^{4}}$	$-\frac{2}{3}\frac{1+{\bar{u}}^{4}{\bar{v}}_{c}^{4}}{{\bar{v}}_{c}^{4}}$	$\frac{2}{{\bar{v}}_{c}^{4}}\frac{{\bar{v}}_{*}^{4}-{\bar{u}}^{4}{\bar{v}}_{c}^{4}}{3{\bar{v}}_{*}+2{\bar{u}}^{4}}$	2∫3∫1−u¯4v¯c4v¯c4	$\frac{2}{{\bar{v}}_{c}^{4}}\frac{{\bar{v}}^{4}-{\bar{u}}^{4}{\bar{v}}_{c}^{4}}{2\bar{v}+{\bar{v}}^{4}+{\bar{u}}^{4}}$
*c*_0_	−2∫v¯24v¯∗+u¯43v¯∗+4u¯4	$-\frac{2}{3{\bar{v}}_{2}^{4}}$	2v¯24v¯∗+u¯43v¯∗+2u¯∫4	$\frac{2}{3{\bar{v}}_{2}^{4}}$	$\frac{2}{{\bar{v}}_{2}^{4}}\frac{{\bar{v}}^{4}}{2\bar{v}+{\bar{v}}^{4}+{\bar{u}}^{4}}$
*d*	$\frac{(2\alpha \;-\;{\bar{S}}_{D0}^{\left(b\right)}){\bar{v}}_{*}}{3{\bar{v}}_{*}\;+\;4{\bar{u}}^{4}}\;+\;\frac{(4\;-\;{\bar{S}}_{D0}^{\left(b\right)}){\bar{u}}^{4}}{3{\bar{v}}_{*}\;+\;4{\bar{u}}^{4}}$	2α − S¯D0(b)∫3∫	$\frac{(4\alpha \;+\;{\bar{S}}_{D0}^{\left(b\right)}){\bar{v}}_{*}}{3{\bar{v}}_{*}\;+\;2{\bar{u}}^{4}}\;+\;\frac{(4\;+\;{\bar{S}}_{D0}^{\left(b\right)}){\bar{u}}^{4}}{3{\bar{v}}_{*}\;+\;2{\bar{u}}^{4}}$	$\frac{4\alpha \;+\;{\bar{S}}_{D0}^{\left(b\right)}}{3}$	$\frac{4\alpha \bar{v}\;+\;4{\bar{u}}^{4}\;+\;{\bar{v}}^{4}{\bar{S}}_{D0}^{\left(b\right)}}{2\bar{v}\;+\;{\bar{v}}^{4}\;+\;{\bar{u}}^{4}}$
*l*	$\frac{(3\alpha -2){\bar{v}}_{*}+2{\bar{u}}^{4}}{3{\bar{v}}_{*}+4{\bar{u}}^{4}}$	α−2∫3∫	$\frac{(6\alpha +2){\bar{v}}_{*}+6{\bar{u}}^{4}}{3{\bar{v}}_{*}+2{\bar{u}}^{4}}$	$2\alpha +\frac{2}{3}$	$\frac{6\alpha \bar{v}+4{\bar{u}}^{4}+2{\bar{v}}^{4}}{2\bar{v}+{\bar{v}}^{4}+{\bar{u}}^{4}}$

### 4.2. Buoyancy

First, consider the effect of negative buoyancy, *β*. Because $S_{D0}$, *b*, *m* and $P_{0}$ are independent of *β*

4.11 $$\frac{\beta }{u}\frac{\partial u}{\partial \beta }=\frac{1}{2}$$

and

**Figure 5.**
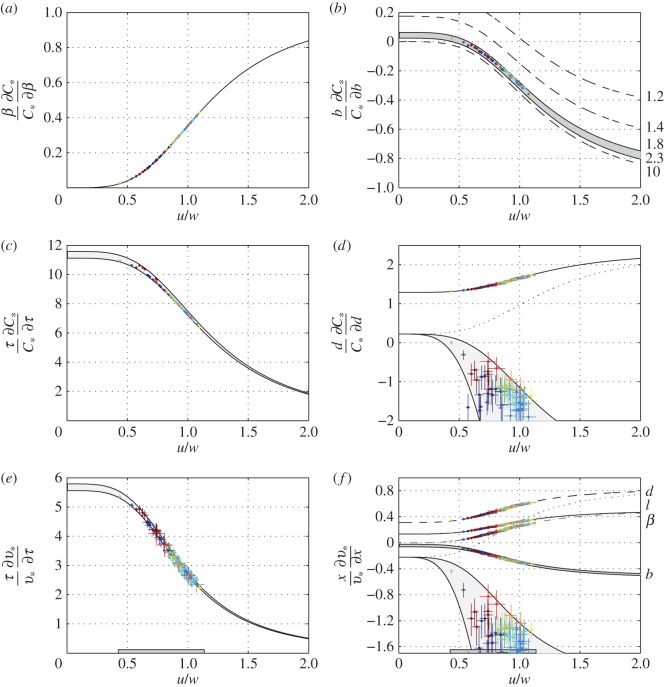
Relative changes in the minimal cost of transport with negative buoyancy (*a*), pectorals span (*b*), body temperature (*c*) and diameter (*d*). Relative changes in the minimal cost of transport speed with body temperature (*e*), pectoral fin span (grey range in (*f*)), negative buoyancy (dashed-dotted line in (*f*)), diameter (broken and dotted lines in (*f*)) and length (solid line in (*f*)). Dotted lines in (*d*) and (*f*) reflect the case when increase in diameter does not yield an increase in the basic metabolic rate; light grey ranges in the same figures mark the composite case where the increase in diameter also increases buoyancy. The borders of these ranges are $B_{l}\text{/}\beta =0.1$ (bottom) and $B_{l}\text{/}\beta =0.5$ (top). The borders of the grey ranges in (*c*) and (*e*) are *τ* = 16°C (top) and *τ* = 28°C (bottom). Relevant range of *u*/*w* values for requiem sharks is indicated by dark grey horizontal bars in (*e*) and (*f*). Crosses mark the individual sharks from electronic supplementary material, S1, tables S2*c* and S2*d*.

4.12 $$\frac{\beta }{w}\frac{\partial w}{\partial \beta }=\frac{\beta }{P_{0}}\frac{\partial P_{0}}{\partial \beta }=0$$

by (4.8), (4.9) and (4.10). The particular derivatives in the first row of table 3 follow these by (4.1), (4.5) and (4.6). All these derivatives are non-negative (figure 5*a,e*); that is, negative buoyancy increases the active metabolic rate, the cost of transport and the speeds at which the minimal values of these parameters are obtained. At the same time, all the derivatives diminish with the ratio *u*/*w*. Indeed, when this ratio becomes smaller than, say 0.7, the cost of transport and the speed at which it is obtained become insensitive to changes in buoyancy (figure 5*a*). As mentioned already, the ratio *u*/*w* can be made small by increasing the buoyancy or/and the body temperature, and, to a lesser extent, the span of the pectoral fins.

### 4.3. Span of the pectoral fins

We assume that an infinitesimal increase Δ*b* in the span of the pectoral fins is accompanied by an increase $\Delta S_{p}=c_{0}\Delta b$ in their projected area, *c*_0_ being the root (proximal, base) chord of the fins. These changes affect induced and parasite drag coefficients alike. The change in the induced drag coefficient is implicitly included in *u*; the change in the parasite drag coefficient is modelled by setting $\Delta S_{D0}=2k_{f}C_{f}\Delta S_{p},$ where *C*_*f*_ is the effective friction coefficient, *k*_*f*_ is the form factor,^9^ and ‘2’ comes from the fact that surface area of the fins is twice their projected area. Thus,

4.13 $$\frac{b}{S_{D0}}\frac{\partial S_{D0}}{\partial b}=\frac{2}{{\bar{v}}_{c}^{4}},$$

where

4.14 $${\bar{v}}_{c}={\left(\frac{S_{D0}}{k_{f}c_{0}bC_{f}}\right)}^{1/4}$$

is a dimensionless parameter that is interpreted below. For a typical requiem shark, ${\bar{v}}_{c}$ varies in a narrow range between 1.8 and 2.2 (see electronic supplementary material, S1, table S2*b*). Thus,

4.15 $$\begin{array}{rl} \frac{b}{P_{0}}\frac{\partial P_{0}}{\partial b} & =0, \end{array}$$

4.16 $$\begin{array}{rl} \frac{b}{u}\frac{\partial u}{\partial b} & =-\frac{1}{2}\left(1+\frac{1}{{\bar{v}}_{c}^{4}}\right) \end{array}$$

4.17 $$\begin{array}{rl} \;\text{and}\;\frac{b}{w}\frac{\partial w}{\partial b} & =-\frac{2}{3{\bar{v}}_{c}^{4}} \end{array}$$

by (4.8)–(4.10) and (4.13). The partial derivatives in the third row of table 3 follow these by (4.1), (4.5) and (4.6). Referring to the last column in this row, increasing the span of the pectoral fins decreases the active metabolic rate and the cost of transport only if

4.18 $$v<u{\bar{v}}_{c}.$$

Large fins are detrimental for high-speed swimming. For a neutrally buoyant fish, for which *u* = 0, increasing the span of the pectoral fins increases the active metabolic rate at any speed other than zero.

Increasing the span of the pectoral fins can reduce the minimal cost of transport only if

4.19 $$v_{*}<u{\bar{v}}_{c}.$$

Condition (4.19) can be elucidated in figure 4*a*. The straight lines in figure 4 can be envisioned as the lines representing $v\text{/}w={\bar{v}}_{c}u\text{/}w$ for different values of ${\bar{v}}_{c}$. With ${\bar{v}}_{c}>1$ (see the paragraph immediately following (4.14)), there is an intersection between the respective line and the line $v_{*}\text{/}w$. The region to the right of the intersection point is the region where increasing the span of the fins reduces the cost of transport, the region to the left of it is where it increases it. In general, however, if *u*/*w* is small (say, smaller than 0.7), all performance parameters become insensitive to the span of the pectoral fins.

### 4.4. Area of the pectoral fins

We assume an infinitesimal increase Δ*c*_0_ of the chord length of the pectoral fins that leaves their span unchanged. It yields an increase $\Delta S_{D0}=4k_{f}C_{f}s\Delta c_{0}$ in the drag area of the shark, *s* being the length (distal margin) of a single fin and the multiplier 4 comes from having two fins and each fin having two sides. Thus,

4.20 $$\frac{c_{0}}{S_{D0}}\frac{\partial S_{D0}}{\partial c_{0}}=\frac{2}{{\bar{v}}_{2}^{4}},$$

where

4.21 $${\bar{v}}_{2}={\left(\frac{S_{D0}}{2k_{f}c_{0}sC_{f}}\right)}^{1/4}={\bar{v}}_{c}{\left(\frac{b}{2s}\right)}^{1/4}.$$

Because b≈2s+d, ${\bar{v}}_{2}>{\bar{v}}_{c}$; and hence ${\bar{v}}_{2}^{4}$ is a large quantity indeed. Thus,

4.22 $$\begin{array}{rl} \frac{c_{0}}{P_{0}}\frac{\partial P_{0}}{\partial c_{0}} & =0, \end{array}$$

4.23 $$\begin{array}{rl} \frac{c_{0}}{u}\frac{\partial u}{\partial c_{0}} & =-\frac{1}{4}\frac{c_{0}}{S_{D0}}\frac{\partial S_{D0}}{\partial c_{0}}=-\frac{1}{2{\bar{v}}_{2}^{4}} \end{array}$$

4.24 $$\begin{array}{rl} \;\text{and}\;\frac{c_{0}}{w}\frac{\partial w}{\partial c_{0}} & =-\frac{1}{3}\frac{c_{0}}{S_{D0}}\frac{\partial S_{D0}}{\partial c_{0}}=-\frac{2}{3{\bar{v}}_{2}^{4}} \end{array}$$

by (4.8)–(4.10) and (4.13). The partial derivatives listed in the fourth row of table 3 follow these three by (4.1), (4.5) and (4.6). All derivatives are of the order of $1\text{/}{\bar{v}}_{2}^{4}$ and hence small. Thus, although minimal active metabolic rate and minimal cost of transport increase with increasing chord of the fins, this increase is ignorable. That being said, increasing the chord decreases the minimal swim speed (see (3.34)), and hence may reduce the minimal active metabolic rate.

### 4.5. Temperature

Again, to start with, a few primitive derivatives are needed. It is assumed that the change in temperature affects only the basic metabolic rate and does not affect any other parameter. Specifically, it does not affect the parasite drag coefficient.^10^ Thus, using (3.14),

4.25 $$\frac{\tau }{u}\frac{\partial u}{\partial \tau }=0$$

and

4.26 $$\frac{\tau }{w}\frac{\partial w}{\partial \tau }=\frac{k_{\tau }}{3\tau }.$$

The partial derivatives listed in the second row of table 3 follow these two by (4.1), (4.5) and (4.6). All derivatives are large and positive, suggesting that lowered body temperatures decrease the active metabolic rate, the cost of transport and the speeds at which the minimal values of these parameters are obtained. For small values of *u*/*w*, a 3°C decrease in the body temperature yields a 12% decrease in the minimal cost of transport (and the active metabolic rate) and a 6% decrease in speed at which the minimal cost of transport is obtained (figure 5*c,e*).

### 4.6. Diameter

In this section, we assess the effect of increasing body diameter on the minimal cost of transport in two *primitive* cases: when the basic metabolic rate increases with mass, and when it remains invariant of it (a composite case is addressed in §4.7). In both cases, it is explicitly assumed that the change in diameter yields no change in buoyancy and no change in span of the pectoral fins, but it does change the drag area and the mass. The change in mass,

4.27 $$\frac{d}{m}\frac{\partial m}{\partial d}=2,$$

follows from (3.1); the change in the drag area is

4.28 $$\frac{d}{S_{D0}}\frac{\partial S_{D0}}{\partial d}=\frac{S_{D0}^{\left(b\right)}}{S_{D0}},$$

where $S_{D0}^{\left(b\right)}$ is the drag area of the body. Tacit assumptions underlying (4.28) are that $S_{D0}^{\left(b\right)}$ is linear in *d*, and that the body diameter has no effect on the drag area of the fins. The ratio ${\bar{S}}_{D0}^{\left(b\right)}=S_{D0}^{\left(b\right)}\text{/}S_{D0}$ on the right-rand side of (4.28) is of the order of 2/3.

In the first case—where an increase in diameter yields an increase in the basic metabolic rate

4.29 $$\frac{d}{P_{0}}\frac{\partial P_{0}}{\partial d}=2\alpha$$

by (4.27) and (3.14), whereas

4.30 $$\frac{d}{u}\frac{\partial u}{\partial d}=1-\frac{1}{4}{\bar{S}}_{D0}^{\left(b\right)}$$

and

4.31 $$\frac{d}{w}\frac{\partial w}{\partial d}=\frac{1}{3}(2\alpha -{\bar{S}}_{D0}^{\left(b\right)})$$

by (4.8), (4.9), (4.27), (4.29) and (4.28). The partial derivatives listed in the fifth row of table 3 follow these two by (4.1), (4.5) and (4.6). All derivatives are positive—increasing body diameter increases the active metabolic rate, the cost of transport and the speeds at which the minimal values of these parameters are obtained (figure 5*d,f*). The second case—where an increase in body diameter does not increase the basic metabolic rate—is obtained from the first by setting *α* = 0; this case is exploited below.

### 4.7. Body conditioning

Based on the previous results, we can address now the composite case where an increase in the body diameter is a consequence of storing low-density lipids, and therefore is accompanied by an increase in buoyancy. Adding a small volume Δ*V* of oil of density $\rho_{l}=(1-B_{l})\rho$ to a body of volume *V* and density $\rho_{b}=(1+\beta )\rho$, yields a change

4.32 $$\frac{\Delta d}{d}=\frac{\Delta V}{2V},$$

in the body diameter, and a change

4.33 $$\frac{\Delta \beta }{\beta }=-\frac{\Delta V}{V}\left(1+\frac{B_{l}}{\beta }\right),$$

in the relative excess density.

The combined effect of diameter and buoyancy on the minimal cost of transport and the speed at which it is obtained are

4.34 $$\frac{\Delta C_{*}}{C_{*}}=\left(\frac{d}{C_{*}}\frac{\text{d}C_{*}}{\text{d}d}\right)\frac{\Delta d}{d}$$

and

4.35 $$\frac{\Delta v_{*}}{v_{*}}=\left(\frac{d}{v_{*}}\frac{dv_{*}}{dd}\right)\frac{\Delta d}{d},$$

where

4.36 $$\frac{d}{C_{*}}\frac{dC_{*}}{dd}=\frac{d}{C_{*}}\frac{\partial C_{*}}{\partial d}+\frac{\beta }{C_{*}}\frac{\partial C_{*}}{\partial \beta }\frac{d}{\beta }\frac{\partial \beta }{\partial d}$$

and

4.37 $$\frac{d}{v_{*}}\frac{dv_{*}}{dd}=\frac{d}{v_{*}}\frac{\partial v_{*}}{\partial d}+\frac{\beta }{v_{*}}\frac{\partial v_{*}}{\partial \beta }\frac{d}{\beta }\frac{\partial \beta }{\partial d}.$$

**Figure 6.**
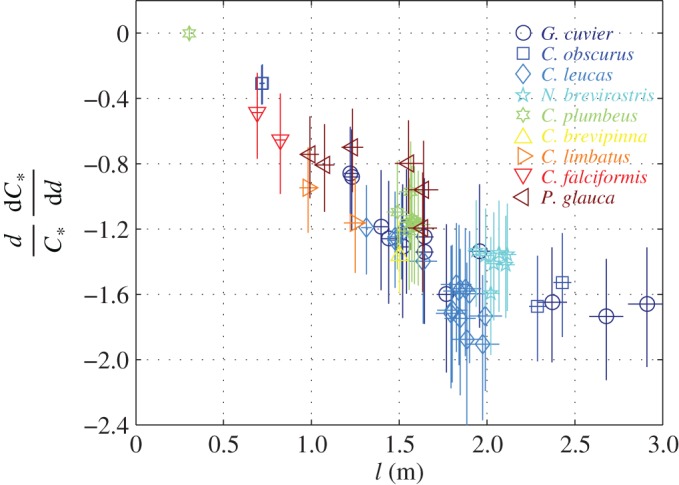
Relative change in the minimal cost of transport with body diameter under the assumption that the addition to the diameter comes from stored lipids. Crosses mark the uncertainty range; relevant numerical values can be found in electronic supplementary material, S1, table S2*c*. The highest point belongs to a pup of *C. plumbeus.* Assumptions: ${\bar{S}}_{D0}^{\left(b\right)}\approx 2\text{/}3$, $B_{l}\in (0.1,0.12)$.

The derivatives $\frac{d}{C_{*}}\frac{\partial C_{*}}{\partial d}$, $\frac{\beta }{C_{*}}\frac{\partial C_{*}}{\partial \beta }$, $\frac{d}{v_{*}}\frac{\partial v_{*}}{\partial d}$ and $\frac{\beta }{v_{*}}\frac{\partial v_{*}}{\partial \beta }$ are found in table 3. Stored lipids do not require energy to be maintained, and therefore, the increase in diameter does not yield an increase in the basic metabolic rate. Consequently, the first derivative has to be accessed with *α* = 0 (see above). The last derivative on the right-hand side of (4.36) and (4.37),

4.38 $$\frac{d}{\beta }\frac{\partial \beta }{\partial d}=-2\frac{\beta +B_{l}}{\beta },$$

follows from (4.33) and (4.32). The derivatives$\frac{d}{C_{*}}\frac{dC_{*}}{dd}$ and $\frac{d}{v_{*}}\frac{dv_{*}}{dd}$ are shown in figures 5*d,f* and 6. In general, $\frac{d}{C_{*}}\frac{dC_{*}}{dd}$ is negative, manifesting the energetic advantage of becoming fatter—the advantage that increases as the animal becomes larger (figure 6). The exception is the pup of *C*. *plumbeus*, for which this derivative practically vanishes. In spite of the diminutive size of the pup, which actually makes it too small to be adequately described by the present theory, this result seems to accord observations of Hussey *et al.* [28]: having the cost of transport insensitive to conditioning, pups can turn stored lipids into growth with no energetic penalty. In general, $\frac{d}{v_{*}}\frac{dv_{*}}{dd}$is negative and large, manifesting that the optimal swim speed is highly sensitive to conditioning, with fat individuals swimming considerably slower than their skinny, but otherwise similar, counterparts.

### 4.8. Length

In assessing the effect of length, we assume that other dimensions of the shark—the diameter of the body and the span and chord of the pectoral fins—increase proportionally to length. Thus,

4.39 $$\frac{l}{m}\frac{\partial m}{\partial l}=3,\;\frac{l}{S_{D0}}\frac{\partial S_{D0}}{\partial l}=2,\;\frac{l}{b}\frac{\partial b}{\partial l}=1,$$

and, consequently,

4.40 $$\begin{array}{rl} \frac{l}{P_{0}}\frac{\partial P_{0}}{\partial l} & =3\alpha , \end{array}$$

4.41 $$\begin{array}{rl} \frac{l}{u}\frac{\partial u}{\partial l} & =\frac{1}{2} \end{array}$$

4.42 $$\begin{array}{rl} \;\text{and}\;\frac{l}{w}\frac{\partial w}{\partial l} & =\alpha -\frac{2}{3} \end{array}$$

by (4.8)–(4.10). The partial derivatives listed in the last row of table 3 follow these three by (4.1), (4.5) and (4.6). All derivatives are positive—increased body length increases the active metabolic rate, the cost of transport and the speeds at which the minimal values of these parameters are obtained. The derivatives increase with increasing value of *u*/*w*. For small values of this parameter, a 10% increase in length yields 25% increase in the minimal cost of transport, but less than 3% increase in speed at which the minimal cost is obtained.

## 5. Pelagic sharks

Among the six major parameters affecting performance of a shark, probably the most intriguing is the span of the pectoral fins. While length, diameter, temperature and negative buoyancy unconditionally increase the cost of transport, and pectoral fins chord hardly affects it, pectoral fins span either increases or decreases it, depending on the ratio of *u*/*w*. If minimizing the cost of transport was the only selective pressure, and if (4.19) would have been unconditionally satisfied, then we would have seen sharks increasing the span of their fins over the generations. This is not the case, and there is large variability in relative size of the pectoral fins within the same species [5].

A partial explanation can be from the hypothesis that selection favours maximizing the difference between energy gained and energy spent, as opposed to just minimizing the energy spent. For reef sharks, which hunt on reefs, large pectoral fins are detrimental to prey capture as the animals have to manoeuvre within the complex three-dimensional structure of the reef itself. At the same time, no essential constraints are placed on the size of pectoral fins of pelagic sharks. In those species, in order to avoid one-directional evolutionary change in the span of the pectoral fins, the cost of transport must be insensitive to it. To this end, the morphology of the shark and its buoyancy should be coordinated in such a way that

5.1 $$\left|\frac{b}{C_{*}}\frac{\partial C_{*}}{\partial b}\right|<\varepsilon ,$$

where *ϵ* is a certain small number that is specified later. For example, *ϵ* = 0.1 implies that a 10% change in the pectorals span yields less than 1% change in the minimal cost of transport. Using table 3 and exploiting (3.25), (5.1) yields

5.2 $$\left|\frac{2}{{\bar{v}}_{c}^{4}}\frac{w^{3}v_{*}-u^{4}({\bar{v}}_{c}^{4}-1)}{3w^{3}v_{*}+2u^{4}}\right|<\varepsilon .$$

Subject to an *a posteriori* verification, this condition is satisfied when u/w is small (figure 5*b*). Consequently, it can be recast as

5.3 $$\frac{1}{w^{4}+u^{4}}\left|w^{4}-u^{4}\left({\bar{v}}_{c}^{4}-\frac{4}{3}\right)\right|<\frac{3}{2}\varepsilon {\bar{v}}_{c}^{4}$$

by (3.26), or, explicitly, as

5.4 $$\left(1-\frac{3}{2}\varepsilon {\bar{v}}_{c}^{4}\right){\left({\bar{v}}_{c}^{4}\left(1+\frac{3}{2}\varepsilon \right)-\frac{4}{3}\right)}^{-1}<\frac{u^{4}}{w^{4}}<\left(1+\frac{3}{2}\varepsilon {\bar{v}}_{c}^{4}\right){\left({\bar{v}}_{c}^{4}\left(1-\frac{3}{2}\varepsilon \right)-\frac{4}{3}\right)}^{-1}.$$

If *ϵ* is greater than $2\text{/}3{\bar{v}}_{c}^{4}$, the left-hand side of (5.4) turns negative and can be replaced by 0 (figure 5*b*). Thus, recalling that ${\bar{v}}_{c}^{4}\gg 1$, we set $\varepsilon =2k_{\varepsilon }\text{/}3{\bar{v}}_{c}^{4}$, where $k_{\varepsilon }\geq 1$, and simplify (5.4) with

5.5 $$\frac{u}{w}<\frac{{\left(1+k_{\varepsilon }\right)}^{1\text{/}4}}{{\bar{v}}_{c}}.$$

In fact, this is an *a posteriori* justification of using (3.26). Essentially, this is a stronger version of (3.41), leading to the same conclusions. Because ${\bar{v}}_{c}$ is practically an invariable parameter (it equals approx. 2), and because $u\text{/}w\propto {\left(\beta l\text{/}b\right)}^{1/2}l^{7/6-\alpha }e^{k_{\tau }/3\tau }$ (see the paragraph following (3.31)), in order to avoid one-directional evolutionary change in the size of pectoral fins, larger pelagic sharks must have larger buoyancy (smaller *β*) or/and larger pectoral fins (larger *b*/*l* ) or/and higher body temperature (larger *τ*). By moving into colder environment, an ectothermic shark must compensate either by increasing the size of its pectoral fins or/and by increasing its buoyancy.^11^

## 6. Discussion

Obligate swimming sharks are faced with multiple selective forces that could act on the evolution of their shape. The goals of adaptation vary but invariably include minimizing the cost of transport and improving the ability to catch prey (manoeuvrability, acceleration, etc.). Clearly, different factors are important for different species based on their requirements, foraging ecology and the environment they reside in.

Our first prediction relates to ontogenetic changes in shape. There should be an increase in swim speed with size, although the magnitude may not be large, which agrees with empirical studies showing larger sharks swimming faster [2,3]. We also predict that size could have an effect on buoyancy across and within the species. As an animal grows, its buoyancy should increase (i.e. *β* should decrease) to maintain swimming performance. The only way to increase buoyancy would be to maintain greater lipid stores in the liver or muscle, or to alter the composition of stored lipids. Combined data of shark size and buoyancy from a range of species show a negative relationship supporting this prediction. Although not a specific measure of buoyancy, several shark species show ontogenetic increases in the proportional size of the liver (hepatosomatic index) [32,33]; all large tunas (which are denser than water and hence obey the same analysis as sharks) have swim bladders, as opposed to smaller ones [34].

Sharks can improve swimming performance by increasing the span of their pectoral fins, which provides two advantages: it lowers the speed that minimizes the cost of transport, and it makes the animal's swimming performance less sensitive to changes in buoyancy. In relation to their negative buoyancy, the span of pectoral fins with pelagic species seems to be of the right dimensions to minimize the cost of transport (the derivative of the cost of transport with respect to span is small); consequently, we observe large variations in the size of pectoral fins within the species. Examples include the blue (*P. glauca*), thresher (*Alopius* spp.) and oceanic whitetip shark (*C. longimanus*) [6]. These species live in the oligotrophic open ocean where food is scarce, patchily distributed and often unpredictable, hence there should be selection for strategies that allow the animal to search as large an area as possible, at minimal energetic cost. There should also be selection for reduced sensitivity in swimming performance with changes in buoyancy to account for periods where food may be hard to find and the animal loses body condition (and buoyancy).

Still, most sharks have pectoral fins that are smaller than would be required to minimize the cost of transport, suggesting a selective pressure against long fins. This is particularly the case for reef-associated species where large fins reduce manoeuvrability (e.g. if the sharks is trying to catch prey on the reef). It is likely that maximizing the ability to catch prey (through improving manoeuvrability in confined spaces) was the driving factor in their evolution, rather than minimizing the cost of transport.

Temperature could have had an effect on the evolution of body shape in sharks. Species residing in cold waters (e.g. temperate or polar species, deep-water sharks) or those regularly operating in cooler waters (e.g. deep-diving species), would have to account for the reduction in metabolic rate (and the associated reduction in optimal swimming speed) by having very low negative buoyancy and/or skinny bodies and/or large pectoral fins. Again, these predictions have support when looking at the morphology of sharks from different habitats and lifestyles. The Portuguese dogfish and the six-gill sharks—deep-water residents [26,27]—maintain near neutral buoyancy by retaining low-density lipids [26]. The blue shark—a deep-diving species [31,35]—has large pectoral fins, low negative buoyancy [9] and a skinny body.

Clearly, multiple forces drive the evolution of body shape in sharks. We believe that our biomechanical energetic approach allows predictions as to how morphology and behaviour (swim speed) are adapted to lifestyle and habitat. Advances in biologging technology should enable the testing of these predictions.

## Supplementary Material

Supplementary 1: Underlying data

## Supplementary Material

Supplementary 2: Wind tunnel experiments

## Footnotes

1 Lift is universally defined as the component of hydrodynamic force in the direction perpendicular to the direction of swimming. Thrust and drag are both defined as the components of hydrodynamic force in the direction of swimming, the former along it, and the latter opposing it. For a self-propelling body—as a swimming shark is—separation between the two is essentially impossible [12]. In this study, we define drag as the respective component of the hydrodynamic force that would have acted on the shark if it were gliding stretched at the same speed and the same body angle. Concurrently, we define thrust as the sum of the respective component of the hydrodynamic force acting on the (actively swimming) shark and the straight-body drag.

2 Part of the lift is undoubtedly generated by the caudal fin [13–17]. This part depends on size and aspect ratio of the pectoral fins, their location relative to the centre of mass, and on the location of the center of buoyancy relative to the center of mass. When these two centers coincide, the lift of the caudal fin counterbalances the pitching moment generated by the shark's body and pectoral fins. Based on the pitching moment measured in the wind tunnel on a model shark that was morphologically similar to requiem sharks addressed here, the lift of the caudal fin will probably not exceed 20% of the total lift generated by the entire shark (electronic supplementary material, S2). An indirect verification that the lift is generated mainly by the pectoral fins can be found in [17]. Effective generation of lift by pectoral fins is stipulated by the Reynolds number being sufficiently high. How high it should be depends on the geometry of the fins, and it cannot be specified *a priori*. As a rough reference, we note that the relation between the pitching moment and lift of the model shark remained unchanged when the Reynolds number (based on the total length) was decreased from 2.1 down to 0.8 × 10^6^. The latter reflects a 1.5 m shark swimming at 0.5 m s^−1^.

3 Referring to equation (4) in electronic supplementary material, S1, logarithmic derivative of the drag coefficient with respect to Reynolds number (Re) can be approximated by ReCD0∂CD0∂Re=−2.58lnRe≈−1.1logRe. With a typical Reynolds number (based on the body length) considered in this study being at least a few hundred thousand (see footnote 2), this estimate suggests that a 10% variation in speed or in characteristic length (each yielding 10% variation in the Reynolds number) should yield 2% variation in parasite drag coefficient. The concurrent change in parasite drag itself (which changes with velocity and length squared) is estimated at 20%.

4 Propulsion efficiency can be defined in quite a few ways, depending on the complementary definition of thrust [12]. In this study, we have defined thrust as the sum of the hydrodynamic force acting on the fish in the direction of swimming and the straight-body drag at the same speed (see footnote 1). In this way, any possible variations in friction between the body and the water are accounted for by the propulsion efficiency. When swimming at high Reynolds numbers, these variations are expected to be small [12], and the propulsion efficiency is expected to be practically the same as the ideal efficiency [20,21].

5 Requiem sharks can be characterized by reaching maximal depth (the distance between dorsal and ventral edges of the swimmer's outline in the sagittal plane) at the caudal end. They are near-anguilliform swimmers, and propel themselves by lateral deformation waves that propagate caudally faster than they swim. The major parameter that determines the (ideal) propulsion efficiency of this type of anguilliform swimmers, is the ratio between the wave speed and the swimming speed [20,21]. Because this ratio can be adjusted by a particular shark, one can plausibly assume that the propulsion efficiency of all requiem sharks is similar, and practically independent of speed. In numerical examples appearing in this study, we have used 0.7, but the results remained essentially the same when it was changed to 0.65 or 0.75.

6 Rendering *v* as the average speed along the course, and *η* as locomotion (rather than propulsion) efficiency [22], equation (3.15) and all its descendants, including (3.22), apply for variable depth and speed (but periodic) swimming.

7 These combinations included body lengths between 0.5 and 3 m, body diameter to length ratio between 0.1 and 0.22, pectoral fins span to body length ratio between 0.3 and 0.8, and pectoral fins chord to body length between 0.07 and 0.13. Other parameters can be found in electronic supplementary material, S1.

8 In this reference, the authors have unequivocally demonstrated that a six-gill shark may have positive, rather than negative, buoyancy, but made no estimate of the buoyancy itself. It can be estimated by several methods. The simplest, perhaps, is by estimating the mechanical power needed to overcome drag $(1\text{/}2)\rho v^{3}SC_{D}$ and equating it with the rate of change of potential energy in unpowered ascent, $-\beta mgv_{a}$ (*v*_*a*_ being the rate of ascent). It turns out to be a negligible few hundredths percent.

9 This is an empirical correction accounting for fin/body interference; its typical value is 1.4. In electronic supplementary material, S1, it appears as *F_n_*.

10 Decreasing water temperature increases viscosity. A change of 3°C reduces the associated Reynolds number by 7% and hence increases the drag coefficient by 1.5% (see footnote 3). We render this effect small when compared with the effect of decreasing temperature on the basic metabolic rate.

11 Moving into colder environment reduces the optimal swim speed (figure 5*e*). In order to generate the same hydrodynamic lift, the shark needs to place the fins at higher (and less efficient) angle relative to the flow. It can reduce the angle by increasing hydrostatic lift (buoyancy) or by increasing the size of the fins.

## Data accessibility

All data underlying this study have been uploaded in two electronic supplementary files.
