# Supplementary material for: Relations between morphology, buoyancy and energetics of requiem sharks
Source: R Soc Open Sci. 2016 Oct 26;3(10):160406. doi: 10.1098/rsos.160406 (PMC5098981; doi:10.1098/rsos.160406)
Supplement: Supplementary 1: Underlying data [file rsos160406supp1.pdf]

SUPPLEMENTARY S1: UNDERLYING DATA

Introduction

The set of data compiled for this study (table S1) includes 58 individuals from 9 species of morphologically similar sharks: *Carcharhinus obscurus*, *C. leucas*, *C. brevipinna*, *C. limbatus*, *C. plumbeus*, *C. falciformis*, *Negaprion breviostris*, *Galeocerdo cuvier*, and *Prionace glauca*, for which in and out of water weights were reported by Baldrige (1970) and Bone and Roberts (1969). We used morphological measurements compiled by Clark and von Schmidt (1965), Bigelow and Schroeder (1948), and Kohler et al (1996), as well as numerous photographs, to fill in data gaps.

Hydrodynamic data was constructed from morphological data using aircraft preliminary design tools (Raymer, 1992) and yet unpublished wind tunnel measurements (supplementary S2). Representative accuracy of preliminary design tools is about 10% (Raymer, 1992), but they were accurate to within a few percent for the model shark tested in the wind tunnel (supplementary S2). Propulsion efficiency was not addressed in this study and it was assumed constant (0.70) for all individuals, regardless of size and species.

Buoyancy

The buoyancy of *C. obscurus*, *C. leucas*, *C. brevipinna*, *C. limbatus*, *C. falciformis*, *C. plumbeus*, *N. breviostris*, and *G. cuvier*, was based on Baldrige (1970); the buoyancy of *P. glauca* was based on Bone and Roberts (1969). The former reference furnished total length and weight of individual sharks, but did not furnish any additional data; the latter reference furnished weight only (no length).

When the weight and total length were given, the girth (or, what is equivalent, the effective body diameter, d) was estimated in two steps. First, the pre-caudal length l was estimated from the total length using statistical relations of Clark and von Schmidt (1965) or

Bigelow and Schroeder (1948). Given *m*, *β*, and l, the diameter was estimated with

$$d = \left(\frac{4}{\pi} \frac{m}{\rho l k_m (1+\beta)} \right)^{1/2}, \tag{1}$$

where the prismatic coefficient k_m was chosen as that of a double ogive, 8/15. When the total length was not given, it was estimated from length-weight relation of Kohler et al (1996). Given the total length, the rest followed by the same paradigm.

Parasite drag coefficient

Drag is contributed by the body of the shark (it will be marked by the index '0') and its 8 fins (they will be marked by the indices '1',…,'8'). Based on equations (12.24) and (12.27) of Raymer (1992),

$$C_{D0} = \sum_{n=0}^{8} \frac{S_n}{S} C_f(\mathrm{Re}_n) F_n I_{n0}, \tag{2}$$

where S_0, …, S_8 are the wet areas of the respective constituents (namely, the contact areas between the corresponding parts and the water); F_0, …, F_8 are form factors (empirical corrections for flow separation) that will be specified below;

$$\mathrm{Re}_n = \frac{\rho v l_n}{\mu}, \tag{3}$$

is the Reynolds number based on the stream-wise dimension of the respective constituent, l_n; $I_{00}, \ldots, I_{80}$ are interference factors; and, finally,

$$C_f(\mathrm{Re}) = \frac{0.455}{(\log_{10} \mathrm{Re})^{2.58}} \tag{4}$$

is the effective friction coefficient. In (3), μ is the viscosity of water. Equation (4) is based on a tacit assumption that the boundary layer is turbulent.

Approximating the shape of the body by double-ogive of length $l_0 = l$ and maximal effective diameter $d_0 = d$,

$$S_0 = \frac{2}{3}\pi l_0 d_0 . \tag{5}$$

The respective form factor is

$$F_0 = 1 + 60\left(\frac{d_0}{l_0}\right)^3 + \frac{1}{400}\left(\frac{l_0}{d_0}\right) \tag{6}$$

by (12.31) of Raymer (1992). $I_{00} = 1.2$ is guessed to account for the drag of the gills.

$S_1,\ldots, S_N$ are, approximately, twice the projected areas of the respective fins. The form factors are

$$F_n \approx 1 + 2\frac{t_n}{l_n} + 100\left(\frac{t_n}{l_n}\right)^3 \tag{7}$$

by Eq. (12.30) of Raymer (1992); $t_1,\ldots,t_N$ are the thicknesses of the fins. $I_{n0} = 1.4$ was set for every $n > 0$ based on the suggestion appearing on page 283 ibid.

Fin dimensions

All requiem sharks have eight fins: a pair of dorsal, a pair of pectoral, a pair of pelvic, one anal, and one caudal. In estimating the drag coefficient, we have assumed that with notable exception of pectoral fins, all other fins share the same relative dimensions specified in table S1. The rationale behind this assumption was that the error, inevitably introduced by this assumption, is smaller than uncertainty of our estimation (tables S2a, S2b). An indirect a posteriori verification of this assumption is furnished by the invariance of the swimming performance to a change in the chord of the pectoral fins (tables S2c, S2d).

Dimensions of pectoral fins are specified in table S2a; they are based largely on Clark and von Schmidt (1965), and partly on Bigelow and Schroeder (1948). Wet area of a fin was computed with

$$S_n = 1.2 s_n c_{0n} , \tag{8}$$

where s_n is the lateral dimension of the nth fin - for the caudal din it is the dorso-ventral

distance between the upper and the lower lobes, for all other fins it is the distance between the distal margin of the fin and the body, whereas c_{0n} is the base dimension of that fin. The characteristic length in (3) was chosen as $c_{0n}/2$.

Span of the pectoral fins was estimated from the body diameter and the length of the fin (the distance between the fin's tip and the body) with

$$b = 1.8s + 0.9d\,. \tag{9}$$

Concurrently, the gross projected area of the pectoral fins (needed for the estimate of the minimal swim speed) was estimated with

$$S_p = 1.08sc_0 + 0.9dc_0\,. \tag{10}$$

Table S1: Fins dimensions. *s* is the lateral dimension of a fin – for the caudal din it is the dorso-ventral distance between the upper and the lower lobes, for all other fins it is the distance between the distal margin of the fin and the body; c_0 is the base of the fin; *l* is pre-caudal length; t/c is the thickness to chord ratio.

parameter	caudal	pectoral	dorsal 1	dorsal 2	anal	pelvic
s/l	0.24	variable	0.13	0.04	0.04	0.06
c_0/l	0.13	variable	0.13	0.08	0.06	0.06
t/c	0.2	0.1	0.1	0.1	0.1	0.1

Maximal lift coefficient

Most of the hydrodynamic lift generated by the shark comes from the pectoral fins. From aerodynamic perspective, these fins can be classified as high-aspect-ratio lifting surfaces. Aircraft preliminary design tools (Raymer, 1992) suggest that if the reference area is chosen as the gross projected area, the maximal lift coefficient of high-aspect ratio surfaces will be determined mainly by the profile underlying these surfaces, and should be practically independent of their planform. We could estimate this maximal lift coefficient, but having obtained it experimentally for a model shark (supplementary S2), we will use the measured value of unity (figure 1). The gross projected area it the area of the pectoral fins S_p will be

defined as their projection onto the coronal plane, including a virtual continuation within the body outlines.

References

Baldridge D.H., 1970, “Sinking factors and average densities of Florida sharks as functions of liver buoyancy,” *Copeia* **4**, pp. 744-754

Bigelow H.B, Schroeder W.C., 1948, “Sharks” in Tee-Van J., Breder C.M, Hildebrand S.F, Parr A.E. and Schroeder W.C. (eds), *Fishes of the Western North Atlantic*, Sears foundation for marine research, New Haven

Bone Q., Roberts B.L., 1969, “The density of elasmobranchs,” *Journal of the Marine Biological Association of the United Kingdom* **49**, pp. 913-937

Clark E, von Schmidt K., 1965, “Sharks of the central Gulf coast of Florida,” *Bulletin of Marine Science* **15**, pp. 13-83

Kohler N.E., Casey J.G., Turner P.A., 1996, *Length-length and length-weight relationships for 13 shark species from the Western North Atlantic,* NOAA Technical Memorandum NMFS-NE-110

Kushmerick M., Davies R., 1969, “The chemical energetics of muscle contraction. II. The chemistry, efficiency and power of maximally working sartorius muscles,” *Proceedings of the Royal Society of London B: Biological Sciences* **174**, pp. 315-347.

Raymer D.P., 1992, *Aircraft design: a conceptual approach*, AIAA educational series, Washington DC, pp 279-281

Table S2a: Compiled data for a few requiem sharks. The data in the shaded columns is taken from Baldrige (1970) and Bones and Roberts (1969). The remaining parameters are based on Clark and von Schmidt (1965), Bigelow and Schroeder (1948), and Kohler et al. (1996). Because all these estimates were not specific, minimal and maximal values for each parameter are provided, based on the respective minimal and maximal relative values found in these references. When only a single value could be found (as, for example, in Bigelow and Schroeder (1948)), the minimal and maximal values were set 10% below and above the provided value. l_t and l are the total and pre-caudal lengths; m_l is the weight of the liver; S_b and S_p are the cross section area of the body and the gross projected area of the pectoral fins. The ratio of pectoral fins length to total length was re-referred to pre-caudal length using the respective average value of total-to-pre-caudal lengths ratio.

	l_t	m	β	m_l	m_l/m	l [m]		c_0/l		s/l		d/l		b/l		S/l^2		S_p/l^2	
species	m	kg		kg		min	max	min	max	min	max	min	max	min	max	min	max	min	max
G. cuvier	2.26	39	0.034	1.76	0.05	1.59	1.70	0.11	0.13	0.15	0.20	0.13	0.15	0.39	0.50	0.014	0.017	0.030	0.047
	1.69	23	0.024	1.45	0.06	1.19	1.27	0.11	0.13	0.15	0.20	0.16	0.18	0.41	0.53	0.020	0.024	0.033	0.050
	1.68	19	0.028	1.26	0.07	1.18	1.26	0.11	0.13	0.15	0.20	0.15	0.16	0.40	0.51	0.017	0.020	0.032	0.048
	1.98	34	0.034	2.42	0.07	1.40	1.49	0.11	0.13	0.15	0.20	0.15	0.17	0.40	0.52	0.018	0.022	0.032	0.049
	2.26	55	0.025	4.15	0.08	1.59	1.70	0.11	0.13	0.15	0.20	0.16	0.18	0.41	0.53	0.020	0.024	0.033	0.050
	1.92	32	0.031	2.49	0.08	1.35	1.44	0.11	0.13	0.15	0.20	0.16	0.17	0.41	0.52	0.019	0.023	0.032	0.049
	2.43	72	0.029	6.26	0.09	1.71	1.82	0.11	0.13	0.15	0.20	0.16	0.18	0.41	0.53	0.021	0.025	0.033	0.051
	2.12	41	0.027	4.26	0.10	1.49	1.59	0.11	0.13	0.15	0.20	0.15	0.17	0.40	0.52	0.018	0.022	0.032	0.049
	2.09	55	0.022	6.21	0.11	1.47	1.57	0.11	0.13	0.15	0.20	0.18	0.20	0.43	0.55	0.026	0.031	0.035	0.053
	3.47	251	0.013	38.3	0.15	2.58	2.78	0.11	0.13	0.15	0.18	0.16	0.18	0.42	0.48	0.021	0.026	0.034	0.047
	3.07	187	0.014	28.5	0.15	2.28	2.46	0.11	0.13	0.15	0.18	0.17	0.19	0.43	0.49	0.023	0.028	0.034	0.048
	3.77	460	0.008	77.7	0.17	2.80	3.02	0.11	0.13	0.15	0.18	0.20	0.22	0.45	0.51	0.030	0.038	0.037	0.051
	2.69	108	0.015	19.1	0.18	1.90	2.02	0.11	0.13	0.15	0.20	0.17	0.19	0.42	0.54	0.024	0.029	0.034	0.052
C. obscurus	3.25	238	0.019	49.4	0.21	2.38	2.48	0.11	0.13	0.22	0.26	0.19	0.20	0.57	0.64	0.028	0.032	0.044	0.060
	3.06	189	0.027	27.2	0.14	2.24	2.33	0.11	0.13	0.22	0.26	0.18	0.20	0.56	0.64	0.026	0.030	0.044	0.060
	1.00	5.5	0.021	1.04	0.19	0.71	0.73	0.11	0.13	0.17	0.21	0.18	0.19	0.47	0.55	0.025	0.028	0.037	0.052
	0.99	5.6	0.021	1.08	0.19	0.70	0.73	0.11	0.13	0.17	0.21	0.18	0.19	0.47	0.55	0.026	0.029	0.037	0.053
C. leucas	2.51	173	0.030	26.0	0.15	1.79	1.98	0.11	0.14	0.22	0.28	0.23	0.26	0.59	0.75	0.040	0.053	0.049	0.075
	2.39	128	0.038	13.0	0.10	1.71	1.88	0.11	0.14	0.22	0.28	0.21	0.24	0.58	0.73	0.034	0.045	0.047	0.072
	2.40	124	0.041	14.0	0.11	1.72	1.89	0.11	0.14	0.22	0.28	0.20	0.23	0.57	0.72	0.032	0.043	0.047	0.071
	2.45	118	0.035	17.0	0.14	1.75	1.93	0.11	0.14	0.22	0.28	0.19	0.22	0.56	0.71	0.029	0.039	0.046	0.070
	2.05	57	0.049	2.40	0.04	1.48	1.55	0.11	0.14	0.24	0.29	0.18	0.20	0.59	0.70	0.027	0.031	0.048	0.068
	2.53	118	0.043	7.60	0.06	1.81	1.99	0.11	0.14	0.22	0.28	0.18	0.21	0.56	0.70	0.026	0.035	0.045	0.068
	1.78	47	0.044			1.28	1.34	0.11	0.14	0.24	0.29	0.21	0.22	0.61	0.72	0.034	0.039	0.050	0.071
	2.00	64	0.037			1.44	1.51	0.11	0.14	0.24	0.29	0.20	0.22	0.61	0.72	0.033	0.038	0.049	0.070

species	l_t	m	β	m_l	m_l/m	l [m]		c_0/l		s/l		d/l		b/l		S/l^2		S_p/l^2	
	m	kg		kg		min	max	min	max	min	max	min	max	min	max	min	max	min	max
	2.65	149	0.039			1.89	2.09	0.11	0.14	0.22	0.28	0.19	0.22	0.56	0.71	0.029	0.039	0.046	0.070
	2.43	104	0.043			1.74	1.91	0.11	0.14	0.22	0.28	0.18	0.21	0.55	0.70	0.026	0.035	0.045	0.068
	2.46	139	0.037			1.76	1.94	0.11	0.14	0.22	0.28	0.21	0.24	0.58	0.73	0.034	0.045	0.047	0.072
	2.63	177	0.042			1.88	2.07	0.11	0.14	0.22	0.28	0.21	0.24	0.58	0.73	0.035	0.047	0.048	0.073
	2.00	59	0.048			1.44	1.51	0.11	0.14	0.24	0.29	0.20	0.21	0.60	0.71	0.030	0.034	0.049	0.069
	2.50	111	0.045			1.79	1.97	0.11	0.14	0.22	0.28	0.18	0.21	0.55	0.70	0.026	0.034	0.045	0.068
	2.18	74	0.047			1.56	1.72	0.11	0.14	0.22	0.28	0.18	0.21	0.55	0.70	0.026	0.034	0.045	0.068
N.brevirostris	2.63	136	0.038	16.1	0.12	1.97	2.07	0.13	0.15	0.23	0.29	0.19	0.20	0.58	0.69	0.027	0.031	0.052	0.075
	2.74	109	0.050	5.50	0.05	2.06	2.16	0.13	0.15	0.23	0.29	0.15	0.17	0.55	0.67	0.019	0.022	0.049	0.071
	2.75	108	0.040	12.8	0.12	2.06	2.17	0.13	0.15	0.23	0.29	0.15	0.17	0.55	0.66	0.019	0.022	0.048	0.071
	2.59	93	0.052	6.20	0.07	1.94	2.04	0.13	0.15	0.23	0.29	0.16	0.17	0.55	0.67	0.019	0.022	0.049	0.071
	2.65	108	0.042	10.9	0.10	1.99	2.09	0.13	0.15	0.23	0.29	0.16	0.18	0.56	0.67	0.021	0.024	0.049	0.072
	2.52	95	0.039			1.89	1.99	0.13	0.15	0.23	0.29	0.16	0.18	0.56	0.67	0.021	0.025	0.050	0.072
	2.69	115	0.033			2.02	2.12	0.13	0.15	0.23	0.29	0.16	0.18	0.56	0.67	0.021	0.025	0.050	0.072
C. plumbeus	2.11	61	0.042	6.50	0.11	1.51	1.64	0.13	0.15	0.23	0.31	0.18	0.20	0.58	0.74	0.024	0.031	0.052	0.079
	2.16	66	0.040	8.80	0.13	1.54	1.68	0.13	0.15	0.23	0.31	0.18	0.20	0.58	0.74	0.025	0.032	0.052	0.080
	2.09	62	0.046	4.40	0.07	1.49	1.62	0.13	0.15	0.23	0.31	0.18	0.20	0.58	0.74	0.025	0.033	0.052	0.080
	2.10	61	0.029	7.60	0.12	1.50	1.63	0.13	0.15	0.23	0.31	0.18	0.20	0.58	0.74	0.025	0.032	0.052	0.080
	2.00	52	0.042	4.80	0.09	1.43	1.55	0.13	0.15	0.23	0.31	0.18	0.20	0.58	0.74	0.024	0.031	0.052	0.079
	2.11	63	0.038	9.80	0.16	1.51	1.64	0.13	0.15	0.23	0.31	0.18	0.20	0.58	0.74	0.025	0.032	0.052	0.080
	2.13	70	0.034	12.9	0.18	1.52	1.65	0.13	0.15	0.23	0.31	0.19	0.21	0.59	0.75	0.027	0.035	0.053	0.081
	0.42	0.4	0.022	0.06	0.14	0.30	0.31	0.13	0.15	0.14	0.17	0.18	0.18	0.42	0.46	0.024	0.026	0.039	0.053
C. brevipinna	1.96	44	0.058	1.86	0.04	1.49	1.51	0.10	0.12	0.19	0.22	0.17	0.17	0.49	0.54	0.022	0.023	0.035	0.047
C. limbatus	1.68	27	0.056	1.43	0.05	1.18	1.32	0.11	0.13	0.19	0.24	0.16	0.19	0.48	0.60	0.021	0.028	0.037	0.056
	1.32	14	0.054			0.93	1.03	0.11	0.13	0.19	0.24	0.17	0.20	0.48	0.60	0.022	0.030	0.038	0.057
C. falciformis	0.94	3.8	0.048	0.16	0.04	0.66	0.72	0.10	0.12	0.16	0.26	0.15	0.17	0.42	0.62	0.018	0.023	0.031	0.052
	1.12	6.5	0.050	0.25	0.04	0.79	0.86	0.10	0.12	0.16	0.26	0.15	0.17	0.43	0.62	0.018	0.023	0.031	0.052
P. glauca	1.43	10	0.033	0.54	0.05	1.02	1.13	0.08	0.10	0.21	0.27	0.13	0.15	0.50	0.61	0.013	0.017	0.028	0.042
	1.32	8.1	0.035	0.48	0.06	0.94	1.04	0.08	0.10	0.21	0.27	0.13	0.15	0.50	0.61	0.013	0.017	0.028	0.042
	2.07	33	0.016	3.35	0.10	1.48	1.63	0.08	0.10	0.21	0.27	0.13	0.15	0.50	0.62	0.013	0.018	0.028	0.042
	1.64	16	0.021	1.60	0.10	1.17	1.30	0.08	0.10	0.21	0.27	0.13	0.15	0.50	0.62	0.013	0.018	0.028	0.042
	2.18	38	0.024	3.20	0.08	1.55	1.72	0.08	0.10	0.21	0.27	0.13	0.15	0.50	0.62	0.013	0.018	0.028	0.042
	2.18	38	0.018	4.10	0.11	1.56	1.72	0.08	0.10	0.21	0.27	0.13	0.15	0.50	0.62	0.014	0.018	0.028	0.042

Table S2b: Parameters estimated from the data of table S2a. C_{D0} and K have been computed from (2) and (3.7) (main text); both are based on S_b as the reference area. P_0 has been estimated by equation (3.14) based on reference temperature specified in the fifth column; it is given in mmol ATP per second; w, u and $v_{\min}(0)$ have been computed with (3.18), (3.17) and (3.34) based on $SC_{L,\max} = S_p$, $\eta_m = 24$ Joule per mmol ATP (Kushmerick and Davies, 1969), and $\eta = 0.7$. $\bar{v}_c$ was calculated with (4.14). v_+, v_*, C_* and P_* were based on the exact expressions found in table 2 in the main text. For a neutrally buoyant fish, $u = 0$, $C_* w / P_0 = P_* / P_0 = 3/2$, $v_* = w$. Large drag coefficient of the blue shark (last species in the table) reflects its small cross section area.

	C_{D0}		K		$\bar{v}_c$		τ	P_0	w m/s		u/w		v_*/w		$v_{\min}(0)$ m/s		v_+ m/s		v_* m/s		$C_* w/P_0$		P_*/P_0	
sp.	min	max	min	max	min	max	°K	mmol/s	min	max	min	max	min	max	min	max	min	max	min	max	min	max	min	max
G.c.	0.23	0.28	0.028	0.051	1.85	2.01	299	0.122	0.56	0.59	0.79	0.89	1.11	1.16	0.44	0.56	0.34	0.40	0.62	0.68	1.68	1.77	1.86	2.05
	0.20	0.23	0.037	0.064	1.89	2.05	299	0.080	0.56	0.58	0.65	0.72	1.05	1.08	0.37	0.47	0.27	0.32	0.59	0.62	1.58	1.63	1.66	1.76
	0.22	0.26	0.032	0.057	1.87	2.03	299	0.069	0.54	0.57	0.67	0.76	1.06	1.09	0.37	0.47	0.28	0.33	0.58	0.62	1.60	1.65	1.69	1.80
	0.20	0.24	0.034	0.060	1.88	2.04	299	0.109	0.57	0.59	0.80	0.89	1.11	1.16	0.46	0.58	0.34	0.40	0.63	0.69	1.68	1.77	1.86	2.05
	0.19	0.22	0.037	0.064	1.89	2.06	299	0.160	0.59	0.61	0.73	0.82	1.08	1.12	0.44	0.55	0.33	0.38	0.64	0.69	1.63	1.70	1.77	1.91
	0.20	0.23	0.035	0.062	1.88	2.05	299	0.104	0.57	0.59	0.76	0.85	1.09	1.13	0.44	0.55	0.33	0.38	0.62	0.67	1.65	1.73	1.80	1.96
	0.18	0.21	0.038	0.066	1.90	2.07	299	0.199	0.60	0.63	0.81	0.91	1.11	1.16	0.49	0.62	0.37	0.43	0.67	0.73	1.69	1.78	1.88	2.08
	0.20	0.24	0.034	0.060	1.88	2.04	299	0.127	0.58	0.60	0.72	0.81	1.08	1.11	0.42	0.53	0.32	0.37	0.62	0.67	1.62	1.69	1.75	1.88
	0.17	0.20	0.043	0.074	1.92	2.10	299	0.160	0.60	0.61	0.70	0.78	1.07	1.10	0.43	0.54	0.32	0.36	0.64	0.68	1.61	1.67	1.72	1.83
	0.16	0.19	0.047	0.065	1.94	2.07	299	0.541	0.66	0.68	0.65	0.69	1.05	1.07	0.42	0.51	0.33	0.36	0.70	0.72	1.59	1.61	1.67	1.71
	0.16	0.18	0.049	0.068	1.95	2.08	299	0.427	0.65	0.66	0.66	0.70	1.06	1.07	0.42	0.51	0.33	0.35	0.69	0.71	1.59	1.61	1.68	1.72
	0.14	0.15	0.059	0.081	2.00	2.16	299	0.878	0.69	0.70	0.56	0.59	1.03	1.04	0.39	0.47	0.29	0.31	0.72	0.73	1.55	1.56	1.59	1.62
	0.17	0.19	0.041	0.071	1.91	2.09	299	0.275	0.62	0.64	0.63	0.70	1.05	1.07	0.40	0.50	0.30	0.34	0.65	0.69	1.57	1.61	1.65	1.73
C.o.	0.16	0.18	0.034	0.045	1.89	2.01	299	0.518	0.65	0.66	0.71	0.75	1.07	1.09	0.48	0.57	0.35	0.38	0.70	0.72	1.62	1.65	1.74	1.79
	0.17	0.19	0.032	0.043	1.88	2.00	299	0.431	0.64	0.66	0.82	0.87	1.12	1.15	0.55	0.65	0.40	0.43	0.72	0.75	1.70	1.75	1.91	2.00
	0.21	0.23	0.041	0.059	1.90	2.03	299	0.025	0.51	0.52	0.52	0.56	1.02	1.03	0.28	0.34	0.20	0.22	0.52	0.54	1.53	1.55	1.57	1.59
	0.21	0.23	0.042	0.060	1.91	2.04	299	0.026	0.51	0.52	0.52	0.55	1.02	1.03	0.28	0.34	0.20	0.22	0.52	0.54	1.53	1.55	1.57	1.59
C.l.	0.15	0.17	0.037	0.065	1.93	2.16	299	0.401	0.62	0.65	0.87	0.98	1.14	1.21	0.60	0.77	0.42	0.47	0.72	0.77	1.75	1.87	2.00	2.25
	0.16	0.18	0.033	0.059	1.89	2.10	299	0.315	0.62	0.64	0.93	1.05	1.18	1.25	0.62	0.80	0.44	0.50	0.73	0.79	1.81	1.96	2.14	2.45
	0.16	0.18	0.032	0.057	1.88	2.08	299	0.307	0.62	0.64	0.96	1.08	1.20	1.27	0.64	0.82	0.45	0.52	0.74	0.81	1.85	2.02	2.21	2.57
	0.16	0.19	0.030	0.053	1.86	2.05	299	0.296	0.62	0.64	0.87	0.98	1.15	1.21	0.57	0.73	0.41	0.47	0.71	0.76	1.75	1.87	2.00	2.26
	0.18	0.21	0.027	0.040	1.84	1.97	299	0.165	0.59	0.60	0.93	1.01	1.18	1.23	0.58	0.71	0.42	0.46	0.69	0.74	1.82	1.92	2.14	2.35
	0.16	0.20	0.027	0.050	1.84	2.02	299	0.296	0.62	0.64	0.95	1.07	1.19	1.27	0.61	0.78	0.44	0.52	0.73	0.80	1.83	2.00	2.18	2.53
	0.17	0.19	0.032	0.047	1.88	2.02	299	0.141	0.58	0.60	0.88	0.95	1.15	1.19	0.56	0.68	0.39	0.43	0.67	0.71	1.76	1.84	2.02	2.19
	0.17	0.19	0.031	0.046	1.87	2.02	299	0.181	0.59	0.61	0.84	0.91	1.13	1.17	0.54	0.66	0.38	0.42	0.67	0.71	1.72	1.79	1.94	2.09

	C_{D0}		K		$\bar{v}_c$		τ	P_0	w m/s		$\frac{u}{w}$		$\frac{v_*}{w}$		$v_{\min}(0)$ m/s		v_+ m/s		v_* m/s		$\frac{C_* w}{P_0}$		$\frac{P_*}{P_0}$	
sp.	min	max	min	max	min	max	°K	mmol/s	min	max	min	max	min	max	min	max	min	max	min	max	min	max	min	max
	0.16	0.19	0.029	0.053	1.86	2.05	299	0.356	0.63	0.65	0.94	1.06	1.19	1.26	0.62	0.80	0.45	0.52	0.75	0.81	1.83	1.99	2.17	2.50
	0.17	0.20	0.027	0.049	1.84	2.02	299	0.267	0.61	0.63	0.94	1.06	1.18	1.26	0.60	0.77	0.44	0.51	0.72	0.79	1.83	1.98	2.16	2.50
	0.15	0.18	0.033	0.059	1.89	2.10	299	0.337	0.62	0.64	0.92	1.03	1.17	1.24	0.62	0.79	0.44	0.50	0.73	0.79	1.80	1.94	2.11	2.41
	0.15	0.17	0.034	0.060	1.89	2.11	299	0.409	0.63	0.65	1.01	1.14	1.23	1.31	0.69	0.88	0.49	0.56	0.78	0.85	1.92	2.11	2.35	2.77
	0.18	0.20	0.029	0.043	1.85	1.99	299	0.170	0.59	0.61	0.94	1.01	1.18	1.23	0.59	0.72	0.42	0.47	0.70	0.74	1.82	1.92	2.15	2.36
	0.17	0.20	0.027	0.049	1.83	2.02	299	0.281	0.61	0.63	0.97	1.09	1.20	1.28	0.62	0.80	0.45	0.53	0.74	0.81	1.86	2.03	2.24	2.61
	0.17	0.21	0.027	0.049	1.83	2.02	299	0.203	0.60	0.62	0.94	1.06	1.19	1.26	0.59	0.76	0.43	0.50	0.71	0.78	1.83	1.99	2.17	2.51
N.b.	0.17	0.20	0.028	0.043	1.81	1.95	299	0.331	0.62	0.64	0.92	1.01	1.17	1.22	0.56	0.69	0.43	0.49	0.72	0.78	1.80	1.91	2.11	2.34
	0.20	0.24	0.021	0.033	1.77	1.89	299	0.277	0.60	0.62	0.99	1.09	1.21	1.28	0.57	0.70	0.45	0.51	0.72	0.80	1.89	2.03	2.29	2.59
	0.20	0.25	0.021	0.033	1.77	1.89	299	0.275	0.59	0.62	0.88	0.96	1.15	1.20	0.50	0.62	0.40	0.45	0.68	0.74	1.76	1.85	2.02	2.22
	0.21	0.25	0.021	0.034	1.77	1.89	299	0.244	0.59	0.62	1.00	1.09	1.22	1.28	0.57	0.70	0.45	0.51	0.72	0.79	1.89	2.03	2.31	2.61
	0.19	0.23	0.023	0.036	1.78	1.90	299	0.275	0.60	0.62	0.91	1.00	1.17	1.22	0.53	0.65	0.41	0.47	0.70	0.76	1.79	1.90	2.09	2.31
	0.19	0.23	0.023	0.036	1.78	1.91	299	0.248	0.60	0.62	0.86	0.95	1.14	1.19	0.50	0.62	0.39	0.45	0.68	0.74	1.74	1.83	1.99	2.18
	0.19	0.23	0.023	0.037	1.78	1.91	299	0.289	0.60	0.63	0.82	0.89	1.12	1.16	0.48	0.59	0.37	0.43	0.67	0.73	1.70	1.77	1.90	2.05
C.p.	0.18	0.23	0.023	0.041	1.78	1.94	299	0.174	0.58	0.60	0.84	0.95	1.13	1.19	0.49	0.63	0.37	0.43	0.65	0.72	1.72	1.83	1.94	2.18
	0.18	0.22	0.023	0.042	1.78	1.94	299	0.186	0.58	0.61	0.82	0.93	1.12	1.18	0.48	0.62	0.36	0.43	0.65	0.71	1.70	1.81	1.91	2.13
	0.18	0.22	0.023	0.042	1.78	1.95	299	0.177	0.58	0.60	0.89	1.00	1.15	1.22	0.52	0.67	0.39	0.46	0.67	0.74	1.76	1.90	2.03	2.31
	0.18	0.22	0.023	0.042	1.78	1.95	299	0.174	0.58	0.60	0.70	0.79	1.07	1.11	0.41	0.53	0.31	0.36	0.62	0.67	1.61	1.68	1.73	1.85
	0.18	0.23	0.023	0.041	1.78	1.94	299	0.153	0.57	0.60	0.83	0.93	1.12	1.18	0.48	0.61	0.36	0.42	0.64	0.70	1.71	1.81	1.92	2.14
	0.18	0.22	0.023	0.042	1.78	1.95	299	0.179	0.58	0.60	0.80	0.91	1.11	1.16	0.47	0.60	0.35	0.42	0.65	0.70	1.69	1.78	1.87	2.08
	0.17	0.21	0.025	0.045	1.79	1.97	299	0.195	0.59	0.61	0.77	0.87	1.10	1.14	0.46	0.59	0.34	0.40	0.64	0.70	1.66	1.75	1.82	2.00
	0.26	0.28	0.056	0.070	1.90	2.00	299	0.003	0.43	0.43	0.43	0.45	1.01	1.01	0.19	0.22	0.14	0.15	0.43	0.44	1.52	1.52	1.53	1.54
C.b.	0.20	0.21	0.036	0.046	1.91	2.02	299	0.134	0.59	0.60	1.07	1.12	1.27	1.30	0.68	0.79	0.48	0.51	0.75	0.79	2.00	2.09	2.54	2.72
C.li.	0.19	0.24	0.030	0.053	1.85	2.02	299	0.091	0.56	0.58	0.96	1.06	1.19	1.26	0.56	0.71	0.41	0.47	0.67	0.74	1.84	1.99	2.20	2.51
	0.19	0.24	0.032	0.055	1.86	2.03	299	0.054	0.54	0.56	0.88	0.98	1.15	1.21	0.50	0.64	0.36	0.41	0.62	0.67	1.76	1.87	2.02	2.26
C.f.	0.23	0.30	0.024	0.056	1.83	2.04	299	0.019	0.48	0.51	0.67	0.81	1.06	1.11	0.37	0.49	0.25	0.31	0.51	0.57	1.60	1.69	1.69	1.88
	0.22	0.28	0.024	0.056	1.84	2.05	299	0.029	0.50	0.53	0.74	0.88	1.08	1.15	0.41	0.56	0.28	0.36	0.54	0.61	1.64	1.76	1.77	2.02
P.g.	0.26	0.34	0.017	0.031	1.85	1.99	291	0.027	0.43	0.45	0.70	0.78	1.07	1.10	0.36	0.46	0.23	0.27	0.46	0.50	1.61	1.67	1.73	1.84
	0.27	0.35	0.017	0.031	1.85	1.99	291	0.022	0.42	0.44	0.70	0.78	1.07	1.10	0.35	0.45	0.22	0.26	0.45	0.49	1.61	1.66	1.72	1.83
	0.23	0.30	0.018	0.032	1.86	2.01	291	0.067	0.47	0.49	0.57	0.63	1.03	1.05	0.31	0.40	0.20	0.23	0.48	0.51	1.55	1.58	1.60	1.65
	0.25	0.32	0.018	0.031	1.85	2.00	291	0.037	0.44	0.47	0.59	0.66	1.04	1.06	0.31	0.40	0.20	0.23	0.46	0.49	1.56	1.59	1.62	1.68
	0.23	0.30	0.018	0.032	1.86	2.01	291	0.075	0.47	0.50	0.71	0.78	1.07	1.10	0.39	0.50	0.25	0.30	0.50	0.55	1.62	1.67	1.73	1.84
	0.23	0.30	0.018	0.032	1.86	2.01	291	0.076	0.47	0.50	0.61	0.68	1.04	1.06	0.34	0.43	0.22	0.26	0.49	0.53	1.57	1.60	1.63	1.70

Table S2c: Parameters estimated from the data of tables S2a and S2b based on table 3 in the main text. First five columns are taken from tables S2a and S2b. The derivative $\frac{d}{C_*}\frac{\mathrm{d}C_*}{\mathrm{d}d}$ has been estimated with $B_l = 0.1$ (see equations (4.36) and (4.38)). It is negative and large, increasing in magnitude with the length of a shark. It suggests that there is an energetic driver of fattening up with increasing length. The derivative $\frac{c_0}{C_*}\frac{\partial C_*}{\partial c_0}$ is universally small, suggesting that the cost of transport of requiem sharks is insensitive to the chord of their pectoral fins. The derivatives $\frac{d}{C_*}\frac{\partial C_*}{\partial d}$, $\frac{l}{C_*}\frac{\partial C_*}{\partial l}$ and $\frac{\tau}{C_*}\frac{\partial C_*}{\partial \tau}$ are universally large, suggesting that the cost of transport of all requiem sharks increases with diameter, length and temperature. The derivatives $\frac{b}{C_*}\frac{\partial C_*}{\partial b}$ and $\frac{\beta}{C_*}\frac{\partial C_*}{\partial \beta}$ increase with *β*, rendering the cost of transport of all near-neutrally buoyant sharks insensitive to relative changes in buoyancy and in relative span of their pectoral fins.

	l_t	m	β	τ	C_* mmol ATP / m		$\frac{\beta}{C_*}\frac{\partial C_*}{\partial \beta}$		$\frac{b}{C_*}\frac{\partial C_*}{\partial b}$		$\frac{c_0}{C_*}\frac{\partial C_*}{\partial c_0}$		$\frac{d}{C_*}\frac{\partial C_*}{\partial d}$		$\frac{d}{C_*}\frac{\mathrm{d}C_*}{\mathrm{d}d}$		$\frac{\tau}{C_*}\frac{\partial C_*}{\partial \tau}$		$\frac{l}{C_*}\frac{\partial C_*}{\partial l}$	
species	m	kg		°K	min	max	min	max	min	max	min	max	min	max	min	max	min	max	min	max
G. cuvier	2.26	39	0.034	299	0.359	0.369	0.19	0.27	-0.22	-0.13	0.03	0.05	1.49	1.57	-1.65	-0.89	8.19	9.05	2.41	2.46
	1.69	23	0.024	299	0.225	0.227	0.10	0.15	-0.11	-0.04	0.03	0.04	1.39	1.44	-1.21	-0.59	9.57	10.09	2.34	2.37
	1.68	19	0.028	299	0.199	0.201	0.11	0.17	-0.12	-0.05	0.03	0.05	1.41	1.46	-1.18	-0.57	9.34	9.93	2.35	2.39
	1.98	34	0.034	299	0.320	0.328	0.19	0.27	-0.23	-0.14	0.03	0.05	1.49	1.57	-1.66	-0.91	8.18	9.02	2.41	2.46
	2.26	55	0.025	299	0.440	0.447	0.15	0.21	-0.17	-0.09	0.03	0.05	1.45	1.51	-1.78	-0.95	8.80	9.51	2.38	2.42
	1.92	32	0.031	299	0.300	0.305	0.17	0.23	-0.19	-0.11	0.03	0.05	1.46	1.53	-1.58	-0.84	8.57	9.33	2.39	2.44
	2.43	72	0.029	299	0.555	0.568	0.20	0.28	-0.24	-0.15	0.03	0.05	1.50	1.58	-2.08	-1.17	8.08	8.92	2.42	2.47
	2.12	41	0.027	299	0.355	0.360	0.14	0.20	-0.16	-0.08	0.03	0.05	1.44	1.50	-1.60	-0.83	8.91	9.60	2.37	2.42
	2.09	55	0.022	299	0.432	0.435	0.13	0.18	-0.15	-0.08	0.02	0.04	1.42	1.48	-1.75	-0.93	9.15	9.76	2.36	2.40
	3.47	251	0.013	299	1.281	1.294	0.10	0.12	-0.09	-0.05	0.03	0.04	1.40	1.42	-2.13	-1.38	9.80	10.05	2.34	2.36
	3.07	187	0.014	299	1.034	1.041	0.11	0.13	-0.09	-0.06	0.03	0.04	1.40	1.42	-2.02	-1.31	9.76	10.01	2.34	2.36
	3.77	460	0.008	299	1.935	1.963	0.06	0.07	-0.04	-0.02	0.02	0.03	1.35	1.36	-2.04	-1.31	10.39	10.54	2.31	2.32
	2.69	108	0.015	299	0.692	0.695	0.09	0.13	-0.09	-0.04	0.03	0.04	1.38	1.43	-1.81	-0.93	9.72	10.19	2.33	2.36
C. obscurus	3.25	238	0.019	299	1.278	1.287	0.14	0.16	-0.12	-0.08	0.03	0.05	1.43	1.46	-1.86	-1.23	9.37	9.67	2.37	2.39
	3.06	189	0.027	299	1.143	1.149	0.21	0.25	-0.20	-0.15	0.04	0.05	1.51	1.55	-2.01	-1.36	8.38	8.79	2.42	2.45
	1.00	5.5	0.021	299	0.075	0.077	0.04	0.06	-0.02	0.01	0.03	0.04	1.34	1.35	-0.44	-0.19	10.54	10.70	2.30	2.31
	0.99	5.6	0.021	299	0.076	0.077	0.04	0.06	-0.02	0.01	0.03	0.04	1.33	1.35	-0.44	-0.19	10.55	10.70	2.30	2.31

species	l_t	m	β	τ	C_* mmol ATP / m		$\frac{\beta}{C_*}\frac{\partial C_*}{\partial \beta}$		$\frac{b}{C_*}\frac{\partial C_*}{\partial b}$		$\frac{c_0}{C_*}\frac{\partial C_*}{\partial c_0}$		$\frac{d}{C_*}\frac{\partial C_*}{\partial d}$		$\frac{d}{C_*}\frac{dC_*}{dd}$		$\frac{\tau}{C_*}\frac{\partial C_*}{\partial \tau}$		$\frac{l}{C_*}\frac{\partial C_*}{\partial l}$	
	m	kg		°K	min	max	min	max	min	max	min	max	min	max	min	max	min	max	min	max
C. leucas	2.51	173	0.030	299	1.107	1.185	0.25	0.33	-0.30	-0.20	0.02	0.04	1.55	1.64	-2.40	-1.41	7.45	8.39	2.45	2.51
	2.39	128	0.038	299	0.918	0.981	0.30	0.39	-0.35	-0.24	0.03	0.05	1.60	1.70	-2.18	-1.30	6.84	7.86	2.49	2.55
	2.40	124	0.041	299	0.915	0.980	0.32	0.42	-0.37	-0.26	0.03	0.05	1.63	1.72	-2.14	-1.30	6.54	7.58	2.50	2.57
	2.45	118	0.035	299	0.836	0.874	0.25	0.34	-0.29	-0.19	0.03	0.05	1.55	1.64	-2.04	-1.18	7.43	8.39	2.45	2.51
	2.05	57	0.049	299	0.511	0.524	0.30	0.36	-0.31	-0.23	0.04	0.06	1.60	1.67	-1.51	-0.97	7.13	7.83	2.49	2.53
	2.53	118	0.043	299	0.879	0.927	0.31	0.41	-0.36	-0.25	0.04	0.06	1.62	1.71	-2.02	-1.21	6.65	7.69	2.50	2.56
	1.78	47	0.044	299	0.427	0.439	0.26	0.32	-0.27	-0.20	0.03	0.05	1.56	1.62	-1.48	-0.93	7.66	8.31	2.46	2.50
	2.00	64	0.037	299	0.524	0.535	0.23	0.28	-0.24	-0.17	0.03	0.05	1.53	1.58	-1.56	-0.97	8.04	8.65	2.43	2.47
	2.65	149	0.039	299	1.037	1.099	0.31	0.40	-0.36	-0.24	0.03	0.05	1.61	1.71	-2.19	-1.32	6.71	7.74	2.49	2.56
	2.43	104	0.043	299	0.798	0.840	0.31	0.40	-0.35	-0.24	0.04	0.06	1.61	1.71	-1.95	-1.16	6.72	7.76	2.49	2.56
	2.46	139	0.037	299	0.968	1.032	0.29	0.38	-0.34	-0.23	0.03	0.05	1.59	1.68	-2.22	-1.32	6.96	7.97	2.48	2.54
	2.63	177	0.042	299	1.229	1.338	0.36	0.46	-0.42	-0.30	0.03	0.05	1.67	1.77	-2.37	-1.48	6.06	7.13	2.53	2.60
	2.00	59	0.048	299	0.523	0.538	0.30	0.36	-0.31	-0.24	0.04	0.05	1.60	1.67	-1.56	-1.00	7.11	7.81	2.49	2.53
	2.50	111	0.045	299	0.853	0.902	0.33	0.42	-0.38	-0.26	0.04	0.06	1.63	1.73	-1.98	-1.20	6.44	7.50	2.51	2.58
	2.18	74	0.047	299	0.623	0.655	0.31	0.40	-0.35	-0.24	0.04	0.06	1.61	1.71	-1.78	-1.05	6.69	7.73	2.49	2.56
N.brevirostris	2.63	136	0.038	299	0.964	0.990	0.29	0.36	-0.30	-0.22	0.04	0.06	1.59	1.66	-1.97	-1.25	7.19	7.96	2.48	2.53
	2.74	109	0.050	299	0.876	0.902	0.35	0.42	-0.36	-0.27	0.05	0.07	1.65	1.73	-1.75	-1.12	6.49	7.32	2.52	2.58
	2.75	108	0.040	299	0.810	0.823	0.26	0.32	-0.26	-0.18	0.05	0.07	1.56	1.63	-1.70	-1.04	7.57	8.32	2.45	2.50
	2.59	93	0.052	299	0.782	0.805	0.35	0.42	-0.36	-0.27	0.05	0.07	1.65	1.73	-1.68	-1.07	6.44	7.28	2.52	2.58
	2.65	108	0.042	299	0.820	0.836	0.28	0.35	-0.29	-0.21	0.05	0.07	1.58	1.66	-1.76	-1.10	7.27	8.04	2.47	2.52
	2.52	95	0.039	299	0.725	0.735	0.25	0.31	-0.25	-0.17	0.05	0.06	1.55	1.61	-1.68	-1.02	7.72	8.45	2.45	2.49
	2.69	115	0.033	299	0.813	0.819	0.21	0.27	-0.21	-0.14	0.05	0.06	1.51	1.57	-1.71	-1.03	8.18	8.85	2.42	2.46
C. plumbeus	2.11	61	0.042	299	0.518	0.530	0.23	0.31	-0.26	-0.15	0.04	0.06	1.53	1.61	-1.53	-0.84	7.71	8.64	2.43	2.50
	2.16	66	0.040	299	0.544	0.555	0.22	0.30	-0.24	-0.14	0.04	0.06	1.51	1.60	-1.55	-0.84	7.87	8.78	2.42	2.48
	2.09	62	0.046	299	0.536	0.554	0.26	0.35	-0.30	-0.19	0.04	0.06	1.56	1.66	-1.57	-0.89	7.26	8.25	2.46	2.52
	2.10	61	0.029	299	0.484	0.487	0.13	0.19	-0.14	-0.06	0.04	0.06	1.43	1.49	-1.33	-0.66	9.06	9.72	2.36	2.41
	2.00	52	0.042	299	0.458	0.466	0.22	0.30	-0.24	-0.14	0.04	0.06	1.52	1.60	-1.45	-0.79	7.85	8.76	2.43	2.49
	2.11	63	0.038	299	0.520	0.528	0.20	0.28	-0.22	-0.13	0.04	0.06	1.50	1.58	-1.51	-0.81	8.08	8.96	2.41	2.47
	2.13	70	0.034	299	0.551	0.560	0.18	0.25	-0.20	-0.11	0.04	0.06	1.47	1.55	-1.54	-0.82	8.39	9.20	2.40	2.45
	0.42	0.4	0.022	299	0.011	0.011	0.02	0.03	0.02	0.03	0.03	0.04	1.31	1.32	-0.04	0.04	10.91	10.96	2.28	2.29
C. brevipinna	1.96	44	0.058	299	0.455	0.465	0.41	0.45	-0.40	-0.35	0.04	0.05	1.72	1.76	-1.59	-1.15	6.17	6.62	2.57	2.60
C. limbatus	1.68	27	0.056	299	0.298	0.309	0.32	0.40	-0.35	-0.25	0.04	0.05	1.62	1.71	-1.47	-0.89	6.70	7.64	2.50	2.56
	1.32	14	0.054	299	0.175	0.180	0.26	0.34	-0.29	-0.20	0.03	0.05	1.56	1.64	-1.22	-0.70	7.44	8.31	2.46	2.51
C. falciformis	0.94	3.8	0.048	299	0.062	0.063	0.11	0.20	-0.16	-0.05	0.03	0.05	1.41	1.50	-0.77	-0.24	8.94	9.92	2.35	2.41
	1.12	6.5	0.050	299	0.094	0.097	0.15	0.26	-0.22	-0.09	0.03	0.05	1.45	1.56	-0.99	-0.37	8.29	9.48	2.38	2.46
P. glauca	1.43	10	0.033	291	0.098	0.100	0.13	0.18	-0.14	-0.07	0.04	0.05	1.43	1.48	-1.09	-0.56	9.39	10.00	2.36	2.40

	l_t	m	β	τ	C_* mmol ATP / m		$\frac{\beta}{C_*}\frac{\partial C_*}{\partial \beta}$		$\frac{b}{C_*}\frac{\partial C_*}{\partial b}$		$\frac{c_0}{C_*}\frac{\partial C_*}{\partial c_0}$		$\frac{d}{C_*}\frac{\partial C_*}{\partial d}$		$\frac{d}{C_*}\frac{\mathrm{d}C_*}{\mathrm{d}d}$		$\frac{\tau}{C_*}\frac{\partial C_*}{\partial \tau}$		$\frac{l}{C_*}\frac{\partial C_*}{\partial l}$	
species	m	kg		°K	min	max	min	max	min	max	min	max	min	max	min	max	min	max	min	max
	1.32	8.1	0.035	291	0.082	0.084	0.13	0.18	-0.13	-0.07	0.04	0.05	1.42	1.48	-1.01	-0.51	9.42	10.02	2.36	2.40
	2.07	33	0.016	291	0.214	0.222	0.06	0.09	-0.05	-0.00	0.04	0.05	1.35	1.38	-1.11	-0.53	10.45	10.78	2.31	2.33
	1.64	16	0.021	291	0.128	0.132	0.07	0.11	-0.06	-0.02	0.04	0.05	1.37	1.40	-0.97	-0.46	10.26	10.65	2.32	2.35
	2.18	38	0.024	291	0.253	0.258	0.13	0.19	-0.14	-0.07	0.04	0.05	1.43	1.48	-1.59	-0.85	9.36	9.96	2.36	2.40
	2.18	38	0.018	291	0.245	0.252	0.08	0.12	-0.07	-0.02	0.04	0.05	1.37	1.41	-1.31	-0.66	10.15	10.57	2.33	2.35

Table S2d: Parameters estimated from the data of tables S2a and S2b based on table 3 in the text. First five columns are the same as in table S2c. The derivative $\frac{d}{v_*}\frac{\mathrm{d}v_*}{\mathrm{d}d}$ has been estimated with $B_l = 0.1$ (see equations (4.37) and (4.38)). The derivatives $\frac{\beta}{v_*}\frac{\partial v_*}{\partial \beta}$, $\frac{l}{v_*}\frac{\partial v_*}{\partial l}$, $\frac{b}{v_*}\frac{\partial v_*}{\partial b}$ and $\frac{c_0}{v_*}\frac{\partial v_*}{\partial c_0}$ are invariably small, suggesting that the optimal swim speed of all requiem sharks is insensitive to length, buoyancy and the size (span and chord) of the pectoral fins. Thus said, the optimal speed slightly increases with length, and decreases with increasing buoyancy (decreasing β) and the span of the pectoral fins. The derivatives $\frac{d}{v_*}\frac{\mathrm{d}v_*}{\mathrm{d}d}$ and $\frac{\tau}{v_*}\frac{\partial v_*}{\partial \tau}$ are invariably large, suggesting that the optimal speed is highly sensitive to temperature (it increases with increasing temperature) and body conditioning (decreasing as the shark fattens up).

species	l_t m	m kg	β	τ °K	v_* m/s min	v_* m/s max	$\frac{\beta}{v_*}\frac{\partial v_*}{\partial \beta}$ min	max	$\frac{b}{v_*}\frac{\partial v_*}{\partial b}$ min	max	$\frac{c_0}{v_*}\frac{\partial v_*}{\partial c_0}$ min	max	$\frac{d}{v_*}\frac{\partial v_*}{\partial d}$ min	max	$\frac{d}{v_*}\frac{\mathrm{d}v_*}{\mathrm{d}d}$ min	max	$\frac{\tau}{v_*}\frac{\partial v_*}{\partial \tau}$ min	max	$\frac{l}{v_*}\frac{\partial v_*}{\partial l}$ min	max
G. cuvier	2.26	39	0.034	299	0.62	0.68	0.16	0.21	-0.25	-0.21	-0.044	-0.027	0.48	0.53	-1.70	-1.15	3.23	3.80	0.25	0.29
	1.69	23	0.024	299	0.59	0.62	0.09	0.13	-0.16	-0.14	-0.040	-0.025	0.41	0.44	-1.47	-0.96	4.18	4.59	0.20	0.23
	1.68	19	0.028	299	0.58	0.62	0.10	0.14	-0.18	-0.15	-0.043	-0.026	0.42	0.46	-1.43	-0.94	4.00	4.46	0.21	0.24
	1.98	34	0.034	299	0.63	0.69	0.16	0.21	-0.25	-0.21	-0.040	-0.024	0.48	0.53	-1.70	-1.17	3.22	3.78	0.25	0.29
	2.26	55	0.025	299	0.64	0.69	0.13	0.18	-0.21	-0.18	-0.039	-0.024	0.45	0.49	-1.87	-1.24	3.63	4.13	0.23	0.26
	1.92	32	0.031	299	0.62	0.67	0.14	0.19	-0.22	-0.19	-0.040	-0.024	0.46	0.51	-1.68	-1.13	3.47	4.00	0.24	0.27
	2.43	72	0.029	299	0.67	0.73	0.17	0.22	-0.25	-0.22	-0.038	-0.022	0.49	0.54	-2.02	-1.38	3.16	3.71	0.26	0.29
	2.12	41	0.027	299	0.62	0.67	0.12	0.17	-0.20	-0.18	-0.041	-0.025	0.44	0.49	-1.73	-1.15	3.70	4.20	0.22	0.26
	2.09	55	0.022	299	0.64	0.68	0.11	0.15	-0.19	-0.16	-0.036	-0.021	0.43	0.47	-1.89	-1.24	3.87	4.33	0.22	0.25
	3.47	251	0.013	299	0.70	0.72	0.09	0.11	-0.14	-0.14	-0.034	-0.024	0.41	0.43	-2.31	-1.68	4.36	4.56	0.20	0.21
	3.07	187	0.014	299	0.69	0.71	0.10	0.11	-0.15	-0.14	-0.033	-0.023	0.41	0.43	-2.21	-1.61	4.33	4.53	0.20	0.22
	3.77	460	0.008	299	0.72	0.73	0.06	0.07	-0.10	-0.10	-0.029	-0.019	0.37	0.38	-2.34	-1.67	4.84	4.98	0.17	0.18
	2.69	108	0.015	299	0.65	0.69	0.08	0.12	-0.15	-0.13	-0.037	-0.022	0.40	0.43	-2.01	-1.28	4.30	4.68	0.19	0.22
C. obscurus	3.25	238	0.019	299	0.70	0.72	0.12	0.14	-0.18	-0.17	-0.040	-0.029	0.44	0.46	-2.01	-1.50	4.03	4.26	0.22	0.24
	3.06	189	0.027	299	0.72	0.75	0.18	0.20	-0.24	-0.23	-0.040	-0.029	0.50	0.52	-2.01	-1.53	3.35	3.62	0.26	0.28
	1.00	5.5	0.021	299	0.52	0.54	0.04	0.06	-0.09	-0.09	-0.039	-0.027	0.36	0.37	-0.84	-0.62	4.98	5.12	0.16	0.17
	0.99	5.6	0.021	299	0.52	0.54	0.04	0.05	-0.09	-0.09	-0.038	-0.027	0.36	0.37	-0.85	-0.62	4.99	5.13	0.16	0.17
C. leucas	2.51	173	0.030	299	0.72	0.77	0.20	0.25	-0.28	-0.24	-0.035	-0.019	0.52	0.57	-2.19	-1.53	2.79	3.36	0.28	0.32
	2.39	128	0.038	299	0.73	0.79	0.23	0.28	-0.31	-0.28	-0.038	-0.021	0.55	0.60	-1.95	-1.40	2.46	3.03	0.30	0.34
	2.40	124	0.041	299	0.74	0.81	0.24	0.29	-0.32	-0.29	-0.039	-0.022	0.57	0.62	-1.89	-1.37	2.31	2.87	0.31	0.35
	2.45	118	0.035	299	0.71	0.76	0.20	0.25	-0.28	-0.25	-0.042	-0.024	0.52	0.57	-1.91	-1.34	2.78	3.35	0.28	0.32
	2.05	57	0.049	299	0.69	0.74	0.23	0.27	-0.30	-0.28	-0.044	-0.030	0.55	0.59	-1.49	-1.14	2.62	3.01	0.30	0.33
	2.53	118	0.043	299	0.73	0.80	0.24	0.29	-0.32	-0.29	-0.043	-0.025	0.56	0.61	-1.82	-1.32	2.36	2.93	0.31	0.35
	1.78	47	0.044	299	0.67	0.71	0.21	0.24	-0.27	-0.25	-0.039	-0.026	0.53	0.56	-1.52	-1.14	2.91	3.30	0.28	0.31

species	l_t	m	β	τ	v_* m/s		$\frac{\beta}{v_*}\frac{\partial v_*}{\partial \beta}$		$\frac{b}{v_*}\frac{\partial v_*}{\partial b}$		$\frac{c_0}{v_*}\frac{\partial v_*}{\partial c_0}$		$\frac{d}{v_*}\frac{\partial v_*}{\partial d}$		$\frac{d}{v_*}\frac{\mathrm{d}v_*}{\mathrm{d}d}$		$\frac{\tau}{v_*}\frac{\partial v_*}{\partial \tau}$		$\frac{l}{v_*}\frac{\partial v_*}{\partial l}$	
	m	kg		°K	min	max	min	max	min	max	min	max	min	max	min	max	min	max	min	max
	2.00	64	0.037	299	0.67	0.71	0.19	0.22	-0.26	-0.23	-0.040	-0.027	0.50	0.54	-1.61	-1.19	3.14	3.52	0.27	0.29
	2.65	149	0.039	299	0.75	0.81	0.24	0.29	-0.32	-0.29	-0.041	-0.024	0.56	0.61	-1.95	-1.40	2.39	2.96	0.31	0.34
	2.43	104	0.043	299	0.72	0.79	0.23	0.29	-0.32	-0.29	-0.044	-0.026	0.56	0.61	-1.78	-1.28	2.40	2.97	0.31	0.34
	2.46	139	0.037	299	0.73	0.79	0.22	0.27	-0.30	-0.27	-0.038	-0.021	0.54	0.60	-1.99	-1.42	2.52	3.09	0.30	0.33
	2.63	177	0.042	299	0.78	0.85	0.27	0.31	-0.34	-0.31	-0.036	-0.020	0.59	0.64	-2.00	-1.47	2.08	2.62	0.33	0.36
	2.00	59	0.048	299	0.70	0.74	0.23	0.27	-0.30	-0.28	-0.041	-0.028	0.55	0.59	-1.53	-1.16	2.61	3.00	0.30	0.33
	2.50	111	0.045	299	0.74	0.81	0.25	0.30	-0.33	-0.30	-0.044	-0.026	0.57	0.62	-1.77	-1.29	2.26	2.82	0.32	0.35
	2.18	74	0.047	299	0.71	0.78	0.24	0.29	-0.32	-0.29	-0.044	-0.026	0.56	0.61	-1.65	-1.20	2.39	2.95	0.31	0.34
N.brevirostris	2.63	136	0.038	299	0.72	0.78	0.22	0.26	-0.30	-0.28	-0.046	-0.031	0.55	0.59	-1.84	-1.36	2.65	3.09	0.30	0.33
	2.74	109	0.050	299	0.72	0.80	0.26	0.30	-0.34	-0.32	-0.052	-0.036	0.58	0.62	-1.61	-1.22	2.28	2.72	0.32	0.35
	2.75	108	0.040	299	0.68	0.74	0.20	0.24	-0.29	-0.27	-0.054	-0.038	0.52	0.57	-1.68	-1.23	2.86	3.31	0.28	0.31
	2.59	93	0.052	299	0.72	0.79	0.26	0.30	-0.34	-0.32	-0.052	-0.036	0.58	0.62	-1.55	-1.18	2.26	2.70	0.32	0.35
	2.65	108	0.042	299	0.70	0.76	0.22	0.26	-0.30	-0.28	-0.051	-0.035	0.54	0.58	-1.69	-1.25	2.69	3.14	0.29	0.32
	2.52	95	0.039	299	0.68	0.74	0.20	0.24	-0.28	-0.26	-0.052	-0.036	0.52	0.56	-1.67	-1.22	2.94	3.39	0.28	0.31
	2.69	115	0.033	299	0.67	0.73	0.17	0.21	-0.26	-0.23	-0.052	-0.036	0.49	0.53	-1.74	-1.25	3.22	3.66	0.26	0.29
C. plumbeus	2.11	61	0.042	299	0.65	0.72	0.19	0.24	-0.28	-0.25	-0.053	-0.032	0.50	0.56	-1.55	-1.09	2.94	3.52	0.27	0.31
	2.16	66	0.040	299	0.65	0.71	0.18	0.23	-0.27	-0.24	-0.053	-0.032	0.50	0.55	-1.59	-1.10	3.03	3.61	0.26	0.30
	2.09	62	0.046	299	0.67	0.74	0.21	0.26	-0.30	-0.27	-0.051	-0.031	0.53	0.58	-1.55	-1.10	2.68	3.27	0.29	0.32
	2.10	61	0.029	299	0.62	0.67	0.12	0.16	-0.20	-0.18	-0.054	-0.033	0.43	0.48	-1.52	-1.01	3.80	4.30	0.22	0.25
	2.00	52	0.042	299	0.64	0.70	0.18	0.23	-0.27	-0.24	-0.053	-0.033	0.50	0.55	-1.51	-1.05	3.02	3.60	0.26	0.30
	2.11	63	0.038	299	0.65	0.70	0.17	0.22	-0.26	-0.23	-0.052	-0.032	0.49	0.54	-1.58	-1.09	3.16	3.73	0.26	0.29
	2.13	70	0.034	299	0.64	0.70	0.15	0.20	-0.24	-0.21	-0.051	-0.031	0.47	0.52	-1.63	-1.10	3.36	3.91	0.24	0.28
	0.42	0.4	0.022	299	0.43	0.44	0.02	0.02	-0.07	-0.07	-0.037	-0.028	0.33	0.34	-0.48	-0.40	5.32	5.37	0.15	0.15
C. brevipinna	1.96	44	0.058	299	0.75	0.79	0.29	0.31	-0.34	-0.33	-0.034	-0.026	0.61	0.63	-1.48	-1.20	2.13	2.35	0.35	0.36
C. limbatus	1.68	27	0.056	299	0.67	0.74	0.24	0.29	-0.32	-0.29	-0.041	-0.025	0.56	0.61	-1.43	-1.06	2.39	2.90	0.31	0.34
	1.32	14	0.054	299	0.62	0.67	0.21	0.25	-0.29	-0.26	-0.041	-0.025	0.53	0.57	-1.30	-0.95	2.79	3.30	0.28	0.32
C. falciformis	0.94	3.8	0.048	299	0.51	0.57	0.10	0.17	-0.20	-0.16	-0.048	-0.026	0.42	0.49	-1.05	-0.64	3.72	4.45	0.21	0.26
	1.12	6.5	0.050	299	0.54	0.61	0.13	0.21	-0.24	-0.19	-0.047	-0.025	0.45	0.53	-1.18	-0.74	3.29	4.11	0.23	0.28
P. glauca	1.43	10	0.033	291	0.46	0.50	0.12	0.15	-0.19	-0.17	-0.048	-0.032	0.43	0.47	-1.33	-0.91	3.97	4.42	0.22	0.25
	1.32	8.1	0.035	291	0.45	0.49	0.11	0.15	-0.19	-0.17	-0.049	-0.032	0.43	0.47	-1.27	-0.87	3.99	4.44	0.22	0.25
	2.07	33	0.016	291	0.48	0.51	0.06	0.08	-0.12	-0.11	-0.049	-0.032	0.37	0.40	-1.44	-0.93	4.79	5.07	0.18	0.19
	1.64	16	0.021	291	0.46	0.49	0.07	0.10	-0.14	-0.12	-0.049	-0.033	0.38	0.41	-1.30	-0.86	4.63	4.96	0.18	0.20
	2.18	38	0.024	291	0.50	0.55	0.12	0.16	-0.20	-0.17	-0.047	-0.031	0.43	0.48	-1.75	-1.17	3.94	4.39	0.22	0.25
	2.18	38	0.018	291	0.49	0.53	0.08	0.10	-0.14	-0.13	-0.048	-0.032	0.39	0.42	-1.59	-1.04	4.54	4.89	0.19	0.21
