# Supplementary material for: Relations between morphology, buoyancy and energetics of requiem sharks
Source: R Soc Open Sci. 2016 Oct 26;3(10):160406. doi: 10.1098/rsos.160406 (PMC5098981; doi:10.1098/rsos.160406)
Supplement: Supplementary 2: Wind tunnel experiments [file rsos160406supp2.pdf]

SUPPLEMENTARY S2: WIND TUNNEL EXPERIMENTS

Materials and methods

The model

The experimental model was a modified morphologically accurate fifth-scale model of a great hammerhead (*Sphyrna mokarran*) [1]. The modification involved the head only. The model is shown in figure S1. It was printed in FullCure720. All fins had NACA0015 profile. The total length of the model was 640 mm. The part of the model that went into the tunnel was 431 mm long, ending at the caudal end of the anal and second dorsal fins. Its maximal cross section area (that was used to obtain the drag and lift coefficients) was 3870 mm^2.

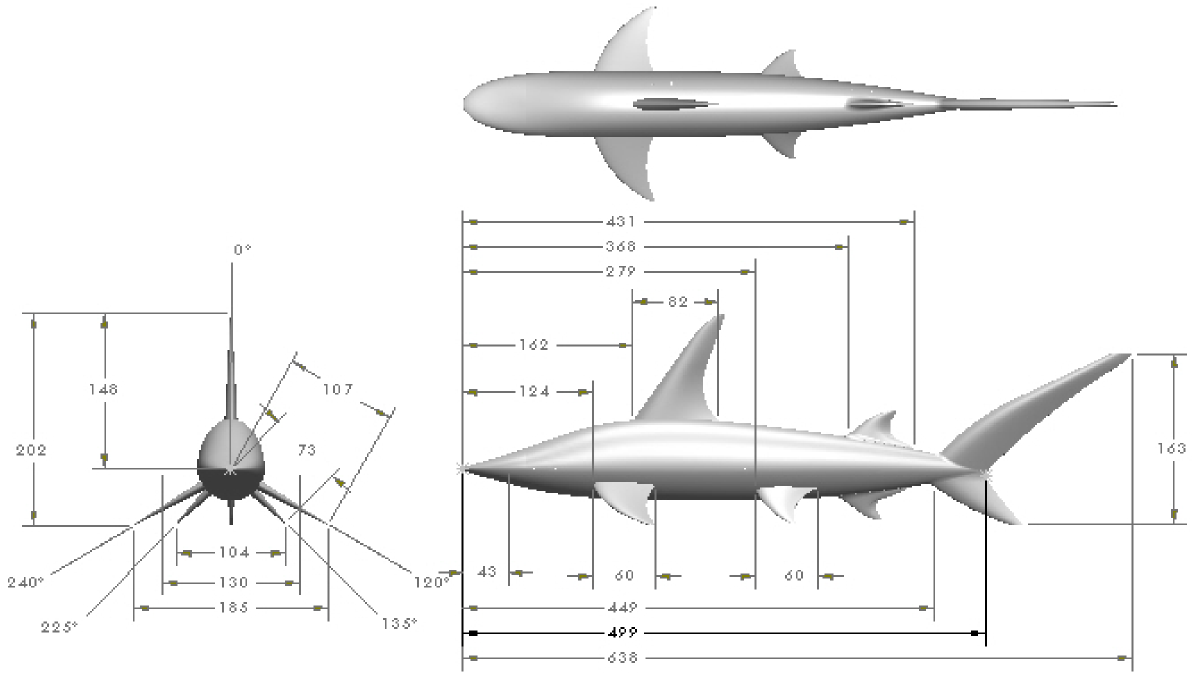

Figure S1: Basic dimensions of the model shark. Center of volume is 227.5 mm from nose.

The wind tunnel

Experiments were conducted at the subsonic wind tunnel of the Faculty of Aerospace Engineering, Technion. This wind tunnel is of the open type, with 1 by 1 by 3 m test section (figure S2). The contraction ratio of the inlet is 23. At 50 m/s, the turbulence intensity across

the test section is, approximately, 0.2%.

The balance

Forces acting on the model were measured with a six-component string balance. Measurement resolution was 1 μv, which is equivalent to approximately 0.42 grams of lift, 0.26 grams of drag, and 2.1 grams-cm of pitching moment. Measurement errors are estimated to be 5 to 10 times larger. During the experiment, the lift and drag were of the order of 10^3 and 10^2 grams, whereas the pitching moment was of the order of 10^4 grams-cm.

The experiment

The single experiment shown below was conducted at 50 m/s; at this airspeed, the corresponding Reynolds number, based on the total length of the model shark (640 mm), is approximately 2 million – the same as the Reynolds number of a 3 m shark swimming at 0.7 m/s in 20°C water. During the experiment, the orientation of the model shark relative to the flow (equivalent to the pitch angle of a free-swimming shark) was changed between minus 15 and plus 15 degrees, at the rate of approximately 0.5 degree a second. The data was acquired at 5 KHz, low-pass filtered at 4 Hz, and block averaged with 500 samples per block.

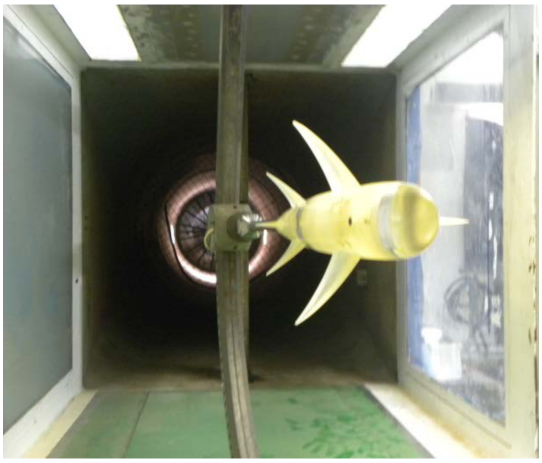

Figure S2: The model shark in the wind tunnel. The flow is along the camera axis. The equivalent of the sea floor is at the left wall. The model moves left-right. The impeller blades of the tunnel are seen at the far end.

Analysis

Lift and drag coefficients

The lift C_L and drag C_D coefficients have been found from the respective forces with

$$C_L = \frac{2L}{\rho v^2 S}, \tag{1}$$

$$C_D = \frac{2D}{\rho v^2 S}, \tag{2}$$

where ρ is the density of air, v is the airspeed, and S is the cross section area of the model (3870 mm squared). The results are shown in figure S3. Flow separation on the pectoral fins starts at the angle of attack of 10 degrees (where the drag coefficient starts to deviate from the curve-fitting parabola), and develops into a full stall at approximately 14 degrees. Drag coefficient predictions of supplementary S1 appear accurate to within a few percent.

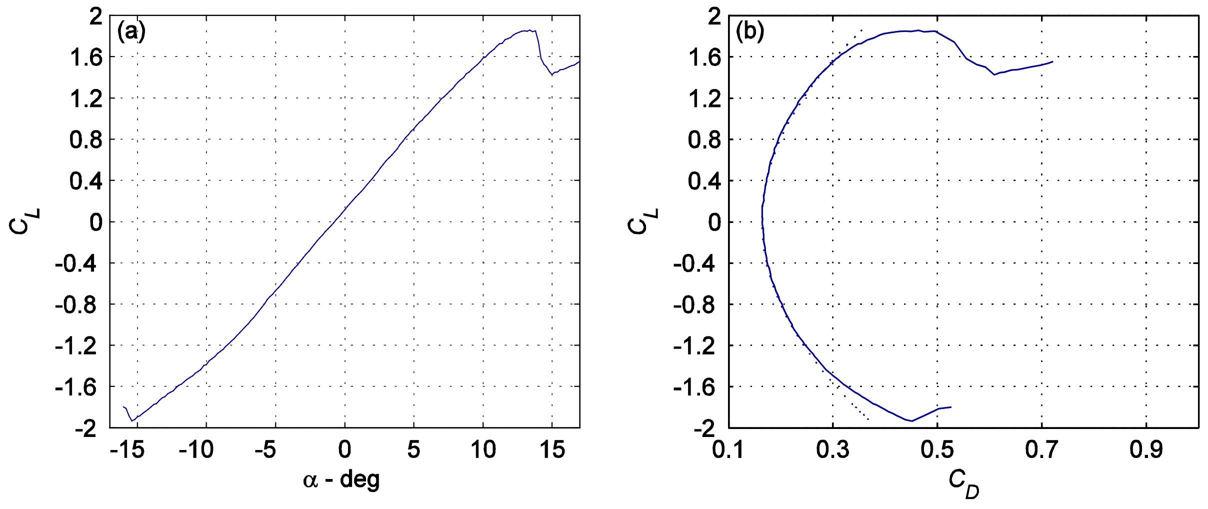

Figure S3: Lift and drag coefficients. α is the angle of attack (the angle between the cranio-caudal axis of the body and the flow direction). Dotted line is a curve-fitting parabola (equation (3.6) in the text) with zero-lift drag coefficient C_{D0} of 0.164 and $k_K = 1.51$. The drag estimates of supplementary S1 suggested $k_K = 1.5$ and C_{D0} =0.162 (no gills drag).

Pitching moment

The pitching moment C_M coefficient has been found from the respective moment M with

$$C_M = \frac{2M}{\rho v^2 S l}, \quad (3)$$

where l is the fork length (499 mm); the moment coefficient shown on figure S4a has been referred to the center of volume of the model, x_{cv}, located 227.5 mm from nose (0.455l). The ratio $-C_M / C_L$ yields the center of pressure x_{cp} relative to x_{cv}:

$$x_{cp} = x_{cv} - \frac{C_M}{C_L}; \quad (4)$$

it is shown on figure S4b. The center of pressure lays within the range of the pectoral fins, indicating that the fins are responsible for most of the lift generated by the (tailless) shark.

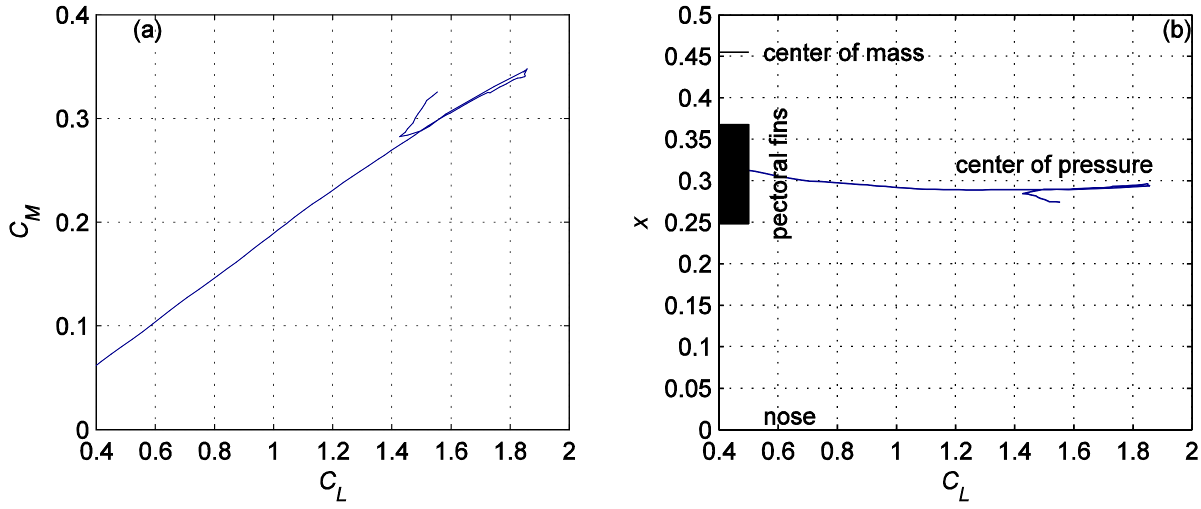

Figure S4: Pitching moment relative to the center of volume (a) and the associated center of pressure relative to the nose (b). Black rectangle in (b) marks the range of the pectoral fins.

Caudal fin contribution

The pitching moment is positive (figure S4a); during swimming it has to be counteracted by the lift of the caudal fin. Under the assumption that the center of volume coincides with the center of mass, the required lift (coefficient) of the fin is

$$C_L^c = \frac{C_M}{x_c - x_{cv}}, \quad (5)$$

where x_c is effective point along the fin where this lift is generated. Consequently,

$$C_L^{tot} = C_L + C_L^c = \left(1 - \frac{x_{cp} - x_{cv}}{x_c - x_{cv}}\right) C_L = \frac{x_c - x_{cp}}{x_c - x_{cv}} C_L \qquad (6)$$

is the total hydrodynamic lift during swimming, and

$$\frac{C_L^c}{C_L^{tot}} = \frac{x_{cp} - x_{cv}}{x_c - x_{cp}} \qquad (7)$$

is the respective part of the caudal fin in this lift. Equation (6) follows (5) by (4); equation (7) follows (6) by (5) and (4). Assuming that x_c is located 1.17*l* from nose (587 mm with the model shark), C_L^{tot} and C_L^c/C_L^{tot} are shown in figures S5a and S5b. The part of the caudal fin is less than 20%.

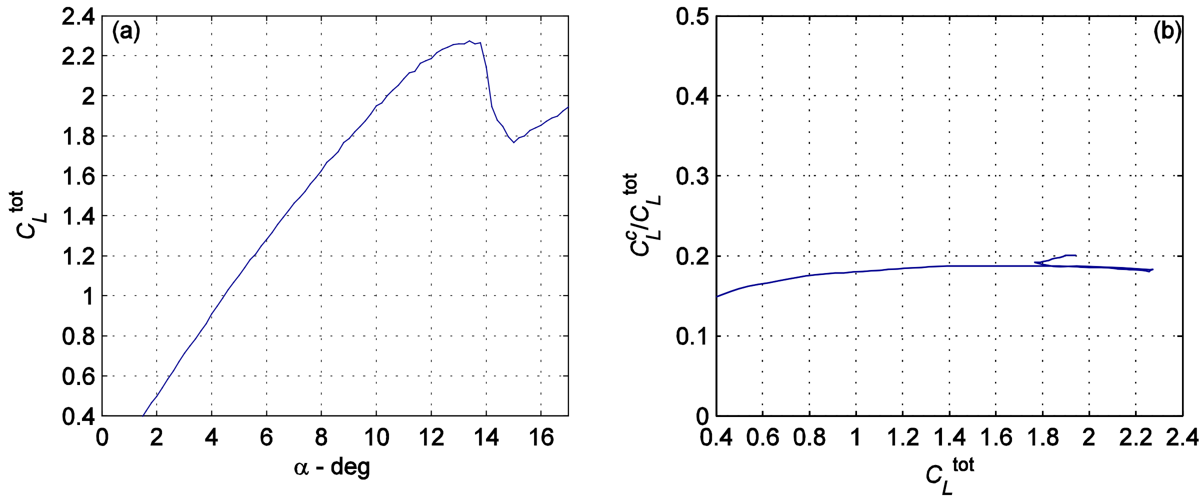

Figure S5: The total hydrodynamic lift of shark during swimming (including the contribution of the caudal fin) (a), and the part of the caudal fin in this lift (b) under the assumption that center of buoyancy coincides with the center of mass.

References

[1] Payne N.L., Iosilevskii G., Barnett A., Fischer C., Graham R.T., Gleiss A.C., Watanabe Y.Y. , 2016, "Great hammerhead sharks swim on their side to reduce transport costs," *Nature Communications* **7**:12289
